# Altered Inflammatory Signature in a *C9ORF72* ‐ALS iPSC‐Derived Motor Neuron and Microglia Coculture Model

**DOI:** 10.1002/glia.70084

**Published:** 2025-09-15

**Authors:** Yujing Gao, Jessica L. Brothwood, Harpreet Saini, Gregory A. O'Sullivan, Carla F. Bento, James M. McCarthy, Nicola G. Wallis, Elena Di Daniel, Brent Graham, Daniel M. Tams

**Affiliations:** ^1^ Astex Pharmaceuticals Cambridge UK

**Keywords:** ALS, coculture, iPSCs, microglia, motor neuron, neuroinflammation, single‐cell RNA sequencing

## Abstract

Amyotrophic lateral sclerosis (ALS) is a complex neurodegenerative disorder involving multiple cell types in the central nervous system. The key pathological features of ALS include the degeneration of motor neurons and the initiation and propagation of neuroinflammation mediated by nonneuronal cell types such as microglia. Currently, the specific mechanisms underlying the involvement of microglia in neuroinflammation in ALS are unclear. Consequently, we generated several human‐induced pluripotent stem cell (iPSC) derived motor neuron and microglia cocultures. We utilized ALS patient‐derived iPSCs carrying a common genetic variant, the hexanucleotide repeat expansion (HRE) in *C9ORF72*, as well as *C9ORF72* knockout (KO) iPSC lines. iPSC‐derived motor neurons and microglia demonstrated expression of cell type‐specific markers and were functional. Phenotypic assessments on motor neurons and microglia in mono‐ and cocultures identified dysfunction in the expression and secretion of inflammatory cytokines and chemokines in lipopolysaccharide (LPS)‐stimulated *C9ORF72* HRE and *C9ORF72* KO microglia. Analysis of single‐cell RNA sequencing data from microglia and motor neuron cocultures revealed cell type‐specific transcriptomic changes. Specifically, we detected the removal of an LPS‐responsive microglia subpopulation, correlating with a dampened inflammatory response in *C9ORF72* HRE and *C9ORF72* KO microglia. Overall, our results support the critical role of microglia‐mediated neuroinflammation in ALS pathology, and our iPSC‐derived models should prove a valuable platform for further mechanistic studies of ALS‐associated pathways.

## Graphical abstract

- Established a *C9ORF72* ALS microglia and motor neuron coculture model.
- Identified an altered inflammatory signature in *C9ORF72* ALS microglia.
- Single‐cell RNA sequencing detected the removal of an LPS responsive microglia subpopulation.

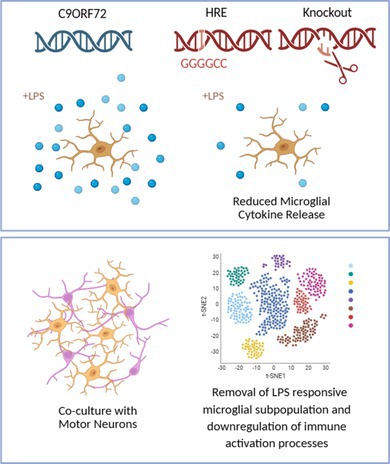

## 1 Introduction

Amyotrophic lateral sclerosis (ALS) is a common form of motor neurone disease (MND), a debilitating and incurable neurodegenerative disorder. The main clinical presentation of ALS is muscle weakness and loss of motor control, mediated by the progressive degeneration of motor neurons (Kiernan et al. 2011). Although ALS primarily occurs sporadically, a genetic etiology is present in approximately 10% of disease cases (Renton et al. 2014). One of the most common genetic causes of ALS is the inclusion of a segment of six repeating nucleotides GGGGCC, or hexanucleotide repeat expansion (HRE), in *C9ORF72*. In *C9ORF72* HRE (C9‐HRE) associated ALS, there is a combination of loss of function (LOF) and gain of function (GOF) mechanisms (Lall and Baloh 2017). First, the HRE is commonly located in the *C9ORF72* promoter region, which can lead to downregulation of *C9ORF72* expression and thus LOF or haploinsufficiency. Second, transcription of HREs leads to the generation of sense and antisense RNA foci, and subsequent initiation of repeat‐associated non‐AUG (RAN) translation results in the generation of dipeptide repeat proteins (DPRs). Both RNA foci and DPRs participate in toxic GOF mechanisms that may contribute to C9‐HRE mediated ALS pathology. Furthermore, the presence of C9‐HRE can be an initiator of protein aggregation and mislocalization, such as for DNA/RNA binding protein TAR DNA‐binding protein 43 (TDP‐43) (Chew et al. 2015; Cook et al. 2020; Ryan et al. 2022). Mislocalization of TDP‐43 from the nucleus to cytoplasm effectively reduces TDP‐43 function and leads to defects in DNA repair and splicing (Mitra et al. 2019).

Currently, the pathological mechanisms underlying ALS are still unclear, although common themes have emerged including dysfunctions in protein degradation, axonal transport, mitochondria, and neuroinflammation (Kiernan et al. 2011; Mead et al. 2023). Microglia are the resident immune cells of the central nervous system (CNS) and are heavily implicated in mediating neuroinflammatory processes in neurodegenerative disorders. In ALS patients and preclinical models, microglia display changes in their morphology and activation state (Clarke and Patani 2020; Graeber 2010). More specifically for C9‐HRE‐associated ALS, mouse models expressing DPRs or reduced levels of *C9ORF72* demonstrated microglial activation and onset of neuroinflammation (O'Rourke et al. 2016; Zhou et al. 2020; Zhu et al. 2020). In addition, microglia derived from C9‐HRE carrying iPSCs have impairments in the immune response to stimulation with *Escherichia coli* derived LPS (Banerjee et al. 2023; Vahsen et al. 2023). Importantly, iPSC‐derived C9‐HRE microglia can be toxic to or increase the vulnerability of motor neurons in cocultures (Banerjee et al. 2023; Vahsen et al. 2023).

To address the potential role of neuroinflammation in the initiation and propagation of neurodegeneration in ALS, we generated and characterized motor neuron and microglia cocultures derived from a panel of commercially available C9‐HRE carrying or *C9ORF72* KO (C9‐KO) iPSC lines. We assessed the LPS‐induced inflammatory profile of monocultured iPSC‐derived microglia, followed by coculture with iPSC‐derived motor neurons. While we observed no overt changes in motor neuron health or function in monoculture or in coculture with microglia, we found that the homeostasis of inflammatory mediators, in particular, those involved in IL‐6 signaling, was dysregulated in C9‐HRE and C9‐KO microglia. These findings were supported by single‐cell RNA sequencing (scRNAseq) data obtained from motor neuron and microglia cocultures, which revealed the loss of an LPS‐responsive microglial subpopulation in C9‐HRE and C9‐KO cocultures. Our motor neuron and microglia coculture models, involving a large panel of C9‐HRE iPSCs, as well as the resultant scRNAseq dataset, should prove a valuable resource for future studies examining the underlying inflammatory mechanisms of ALS and aid in the development of novel therapeutics.

## 2 Methods

### 2.1 iPSC Culture

In this study, we utilized iPSC lines derived from four ALS patients carrying C9‐HRE, an isogenic control line generated through CRISPR/Cas9 genome editing, and three iPSC lines derived from two sex‐ and age‐matched controls. Further, two iPSC lines with C9‐KO were generated through CRISPR/Cas9 genome editing from two control cell lines. Details of cell lines can be found in Table S1. iPSCs were cultured in mTeSR Plus media (100‐0276, StemCell Technologies) on Growth Factor Reduced Matrigel Basement Membrane Matrix (356230, Corning); medium changes were performed daily. Cells were passaged using 0.5 mM EDTA and cryopreserved in CS10 freezing medium (7930, StemCell Technologies). Differentiations were performed within 10 passages from the initial thawing of iPSCs. All iPSC cultures tested negative for mycoplasma using MycoStrip Mycoplasma Detection Kit (rep‐mys‐50, InvivoGen).

### 2.2 Generation of C9‐KO Clones

To generate C9‐KO clones, CS29iALS‐C9n1.ISO2RB4 iPSCs were transduced with Edit‐R predesigned all‐in‐one Lentiviral sgRNA vectors according to the manufacturer's instructions (VSGH12179‐249566259/249380443/249615915, Horizon Discovery). After 72 h, transduced cells were dissociated using Accutase (07920, StemCell Technologies), isolated by EGFP expression using the FACSMelody Cell Sorter (BD Biosciences), and recovered in mTeSR Plus media supplemented with CloneR (05888, StemCell Technologies) until single cells formed distinct colonies. Colonies were expanded for cryopreservation and Sanger sequencing using primers: forward 5′‐TGGGCTCCAAAGACAGAACA‐3′, reverse 5′‐ATATGTGCTGCGATCCCCAT‐3′. Positive clones were further validated by western blotting analysis using C9ORF72 polyclonal antibody (25757‐1‐AP, Proteintech). The undifferentiated status was confirmed by immunocytochemistry using the markers SSEA‐1 (41‐1200, Invitrogen), TRA‐1‐60 (MAB4770, R&D) and NANOG (AF1997, R&D). Pluripotency of newly generated clones was confirmed by immunocytochemistry using the Human Pluripotent Stem Cell Functional Identification Kit (SC027B, R&D). Karyotyping was performed on 20 cells per clone to confirm the lack of genetic aberrations (Cell Guidance Systems, Cambridge, UK). One clone carrying a LOF mutation in exon one of *C9ORF72* (NM_001256054.3:c.200dup) was selected for further study.

### 2.3 iPSC Differentiation to Motor Neurons

iPSCs were differentiated into motor neurons using an adapted protocol (Shi et al. 2018). Briefly, neural induction media was composed of 1:1 DMEM‐F12/Neurobasal (31331‐028/21103‐049, Gibco), 0.5× N‐2 (17502‐048, Gibco), 0.5× B‐27 (17504‐044, Gibco), 1× GlutaMAX (35050‐038, Thermo Scientific), 1× Penicillin‐Streptomycin (15140‐122, Gibco), and 0.1 mM Ascorbic acid (72132, StemCell Technologies). iPSCs were dissociated using Accutase and seeded onto Matrigel‐coated plates (days in vitro [DIV] 0). After 24 h, cell culture media was changed to neural induction media supplemented with 3 μM CHIR99021 (4423, Tocris), 2 μM DMH1 (4126, Tocris), and 2 μM SB431542 (1614, Tocris) for 6 days (DIV 6), then in neural induction media supplemented with 1 μM CHIR99021, 2 μM DMH1, 2 μM SB431542, 0.1 μM retinoic acid (72262, StemCell Technologies), and 0.5 μM Purmorphamine (4551, Tocris) for 6 days (DIV 13). Cells were dissociated using Accutase and seeded onto ultra‐low adherence 96‐well plates (7007, Corning) to form suspended neurospheres in neural induction media supplemented with 0.5 μM retinoic acid and 0.1 μM Purmorphamine. After 6 days (DIV 19), neurospheres were dissociated using Accutase and seeded onto poly(ethyleneimine) (181978, Sigma) and Laminin (L2020, Sigma) coated plates for maturation in neural induction media supplemented with 1 μM Retinoic Acid, 1 μM Purmorphamine, 0.1 μM Compound E (6476, Tocris), 5 ng/mL BDNF (78005, StemCell Technologies), 5 ng/mL GDNF (78058, StemCell Technologies) and 5 ng/mL CNTF (78010, StemCell Technologies). Henceforth, half medium changes were performed every 2 to 3 days.

### 2.4 iPSC Differentiation to Microglia

iPSCs were differentiated into microglia using an adapted protocol (Haenseler et al. 2017). Briefly, iPSCs were dissociated using Accutase and seeded onto ultra‐low adherence 96‐well plates to form embryoid bodies (EBs) in mTeSR Plus media supplemented with 20 ng/mL SCF (300‐07, PeproTech), 50 ng/mL VEGF (100‐20A, PeproTech) and 50 ng/mL BMP4 (314‐BP R&D). After 4 days, EBs were transferred to T75 flasks in X‐VIVO 15 media (BE02‐060F, Lonza) supplemented with 100 ng/mL M‐CSF (300‐25, PeproTech), 25 ng/mL IL‐3 (200‐03, PeproTech), 1× GlutaMAX, and 50 μM 2‐mercaptoethanol (31350‐010, Gibco), with medium removed and replaced weekly. After approximately 1 month, microglia precursors emerging in the supernatant were harvested and matured into microglia by plating onto tissue culture‐treated plates in Advanced DMEM/F‐12 (12634028, Gibco) supplemented with 100 ng/mL IL‐34 (200‐34, PeproTech), 10 ng/mL M‐CSF, 0.5× N‐2, 1× GlutaMAX, and 50 μM 2‐mercaptoethanol. Unless stated otherwise, microglia were primed with 5 ng/mL LPS (L4391, Sigma) for 16 h before collection.

### 2.5 Coculture of iPSC Derived Motor Neurons and Microglia

Motor neuron cultures were matured for approximately 14 days (DIV 30+) before coculturing with microglia. Microglia precursors were harvested, resuspended in coculture media composed of neuron maturation media supplemented with 100 ng/mL IL‐34 and 10 ng/mL M‐CSF, and added to motor neuron cultures at a 1:1 ratio. Cocultures were maintained for approximately 14 days, with half medium changes performed every 2 to 3 days.

### 2.6 Multielectrode Array (MEA)

Spontaneous neuronal activity of motor neuron cultures was measured on 48‐well CytoView MEA plates (M768‐tMEA‐48B, Axion Biosystems) using the Maestro Pro system (Axion Biosystems). Neuronal cultures were supplemented with rat astrocytes (4586, Sartorius) at half the density of neurons and treated with a cocktail of mitotic inhibitors composed of 2.5 μM Cytosine β‐d‐arabinofuranoside (C1768, Sigma), 8 μg/mL (+)‐5‐Fluoro‐2′‐deoxyuridine (227601000, Thermo Scientific) and 28 μg/mL Uridine (140770010, Thermo Scientific) for the initial 48 h. MEA recordings were performed for 5 min using the Neural + Viability configuration, and analysis was performed using the AxIS Navigator software v3.6.2.2 and Neural metric tool v3.2 (Axion Biosystems).

### 2.7 Phagocytosis Assay

Unstimulated or LPS‐primed microglia cultures plated on 96‐well PhenoPlate (6055302, PerkinElmer) were treated with pHrodo Green *E. coli* or Zymosan Bioparticles (4616/4618, Sartorius) and imaged every 30 min for 6 h at ×20 magnification using the Incucyte SX5 Live‐Cell Imaging and Analysis Instrument (Sartorius). Ten micromolar Cytochalasin D (C2618, Sigma) pretreatment for 6 h was included as a control. Analysis was performed on the Incucyte 2022B software (Sartorius), background fluorescence of bioparticles was removed from raw data, then normalized to confluence (%).

### 2.8 Quantification of Secretory Inflammatory Cytokines

Cell culture medium from unstimulated microglia, LPS‐primed microglia or cocultures was collected for downstream immunoassays and performed according to the manufacturer's instructions. Immunoassays included the human inflammation antibody array membrane (ab134003, Abcam), Mesoscale Discovery (MSD) V‐Plex Proinflammatory Panel 1 Human kit (K15049D‐1, MSD), human IL‐6 ELISA Kit (ab178013, Abcam), human TNF alpha ELISA Kit (ab181421, Abcam), and human IL‐10 ELISA Kit (ab46034, Abcam). Data are presented as fold change or log_2_‐transformed fold change compared to the average in untreated samples of the corresponding cell line (pg/mL).

### 2.9 Quantification of Poly(GP) Levels

Motor neuron or microglia cultures were lysed in RIPA buffer (89900, Thermo Scientific) containing 1× cOmplete Mini Protease Inhibitor Cocktail (11836153001, Roche) and 2% SDS (BP2436200, Fisher Scientific). Protein lysates were sonicated at 30% AMP for three times 10 s, centrifuged at 17,000*g* for 20 min at 16°C, and quantified using the Bicinchoninic acid assay (23225, Thermo Scientific). Samples were adjusted to 0.5 mg/mL concentration, and poly(GP) levels were determined using the MSD immunoassay as previously described (Vahsen et al. 2023).

### 2.10 RNA Extraction and Quantitative Real Time PCR (qRT‐PCR)

RNA was extracted using the RNeasy Micro Kit (74004, Qiagen) and the quality and quantity were determined on NanoDrop (ThermoFisher). cDNA was generated from 500 ng of RNA using the QuantiTect Rev. Transcription Kit (205311, Qiagen). Quantitative real‐time PCR was performed using predesigned TaqMan assays and TaqMan Fast Advanced Master Mix (4444964, Applied Biosciences) on the ViiA 7 real‐time PCR system (ThermoFisher). Relative quantification of gene expression was performed using the 2^−ΔΔ*C*t^ method and presented as log_2_‐transformed fold change. Where 2^−ΔΔ*C*t^ is indicated, raw data were normalized to the *GAPDH* housekeeping gene, followed by average expression in corresponding stem cells. Where 2^−Δ*C*t^ is indicated, raw data were normalized to *GAPDH* only. TaqMan assays (Applied Biosciences) include *MAP2* (Hs00258900_m1), *MNX1* (Hs00907365_m1), *CHAT* (Hs00758143_m1), *ITGAM* (Hs00167304_m1), *C9ORF72* (Hs00376619_m1), *TARDBP* (Hs00606522_m1), *GAPDH* (Hs99999905_m1), and the TaqMan Array Human IL‐6 Pathway consisting of a panel of 92 genes (4414149, Applied Biosciences).

### 2.11 scRNAseq

Unstimulated or LPS‐primed cocultures were dissociated using Accutase and resuspended in DMEM/F‐12 supplemented with 0.04% BSA and 10 μM Y‐27632 (72304, StemCell Technologies). Cells were further dissociated by gentle pipetting and filtered through a 35 μm cell strainer (100‐0087, StemCell Technologies) before suspension in PBS (−/−) supplemented with 0.04% BSA and 10 μM Y‐27632. Each sample is composed of approximately 30,000 cells pooled from three independent differentiations. Library preparation was performed using the Chromium Controller platform (10× Genomics) and paired‐end sequencing performed using the NovaSeq6000 platform (Illumina) at 25,000 read depth.

FASTQ files were processed using 10× Genomics Cell Ranger v7.1.0 count (Zheng et al. 2017) and cellbender v7.1.0 (Fleming et al. 2023), then read into R v4.3.0 (R Core Team 2021) using Seurat package v5.0.3 (Hao et al. 2024) and Read_CellBender_h5_Mat from scCustomize v2.1.2 (Marsh et al. 2024). Cells were quality controlled through manual exclusion of outlier cells based on the dataset distribution. Specifically, mitochondrial percentage > 25%, log_2_(count) < 10 for KO/HRE LPS treated, and log_2_(nCount) < 11 for all control and KO/HRE untreated samples, log_2_(nCount) > 16, log_2_(nFeature) < 9, and log_2_(nFeature) > 13.5. Data was then processed using Seurat: log normalization using a scale factor of 10,000, variable features via vst (*n* = 2000), and scaled across all features using default parameters. Principal component analysis (PCA) was run up to 50 PCs, and the optimal number of PCs was selected as 8 using findPC v1.0 (Zhuang et al. 2022) perpendicular line method. Data were visualized via UMAP (uwot v0.1.16), a shared nearest neighbor (SNN) graph was constructed, then Louvain clusters were determined at resolution 0.4. Doublets were then removed using scDblFinder v1.16.0 (Germain et al. 2021). Cell type clusters were identified for neurons and microglia based on the expression of known cell‐type marker genes. The average expression across an inflammatory gene set (*IL‐1A, IL‐1B, CXCL8, TLR2, RIPK2*, and *IL*‐*6*) was plotted using Seurat's AddModuleScore function with default parameters. Differential expression analysis was conducted on genes expressed in a minimum fraction > 0.01 using Seurat's FindMarkers Wilcoxon rank sum test method, returning all genes (logfc.threshold = 0, only.pos = *F*). Gene set enrichment analysis (GSEA) was performed across gene ontology (GO) terms by providing significant hits (adj. *p* < 0.001) ranked by average log fold change * −log_10_(*p* value) as input to the function gseGO in clusterProfiler v4.10.1 (Wu et al. 2021), with Benjamini and Hochberg *p* value adjustment method and minimum and maximum geneset size 100 and 500, respectively. Plots were produced using the simplify function at cutoff 0.7.

### 2.12 Immunofluorescence Imaging

Cells were plated on 96‐well PhenoPlates, fixed for 10 min at room temperature using 4% paraformaldehyde (43368, Alfa Aesar), and incubated in blocking buffer composed of 1% BSA, 0.1% Triton X‐100 (T9284, Sigma) diluted in PBS (−/−) for 1 h at room temperature. Cells were rinsed in PBS (−/−) twice before incubating in primary antibodies diluted in blocking buffer overnight at 4°C. Cells were then washed in PBS (−/−) twice before incubating in Alexa Fluor secondary antibodies (Invitrogen) and Hoechst nucleic acid stain (62249, Thermo Scientific) diluted in blocking buffer for 30 min at room temperature. Finally, cells were rinsed in PBS (−/−) twice and stored in PBS (−/−) until imaging on the Opera Phenix High‐Content System (PerkinElmer). Analysis was performed using Harmony software (PerkinElmer), with approximately 50 cells assessed per differentiation. Primary antibodies utilized include anti‐β‐III tubulin (MAB1195, R&D), anti‐MNX1 (ABN174, Sigma), anti‐choline acetyltransferase (AB144P, Sigma), anti‐IBA1 (17198, Cell Signaling Technology), anti‐PU.1 (ab88082, Abcam), and anti‐TDP‐43 (89789, Cell Signaling Technology).

### 2.13 Western Blotting

Protein was extracted from whole cells using MSD Tris Lysis Buffer (R60TX‐3, MSD) containing 1× cOmplete Mini Protease Inhibitor Cocktail and 1× PhosSTOP Phosphatase Inhibitor Cocktail (4906845001, Roche). Protein concentration was determined by Bicinchoninic acid assay. 10‐20 μg of protein diluted in NuPAGE LDS Sample Buffer (NP0007, Invitrogen) and 200 mM DTT were loaded on NuPAGE 4%–12% Bis‐Tris mini protein gels (NP0335PK2, Invitrogen). Gels were run in NuPAGE MES SDS Running Buffer (NP0002, Invitrogen) at 150 V and transferred to 0.2 μm nitrocellulose membranes (LC2000, Invitrogen) in NuPAGE Transfer Buffer (NP0006, Invitrogen) containing 20% MeOH at 30 V. Subsequently, membranes were blocked in 5% skim milk, incubated with primary antibodies diluted in 2.5% BSA overnight at 4°C, then incubated with IRdye‐conjugated secondary antibodies (LI‐COR Biosciences) for 30 min at room temperature. Membranes were imaged and quantified using the Odyssey CLx system (LI‐COR Biosciences); raw data were normalized to β‐actin signal intensity. Primary antibodies utilized include anti‐C9ORF72 (25757‐1‐AP, Proteintech), anti‐TDP‐43 (89789, Cell Signaling Technology), and anti‐β‐Actin (A5441, Sigma).

### 2.14 Statistical Analysis

Statistical analyses were performed in GraphPad Prism 9.0.2 (San Diego, California USA). The statistical tests used and definition of significance are detailed in individual figure legends. Unless otherwise noted, datasets containing multiple variables were analyzed using two‐way ANOVA with Sidak's post hoc multiple comparisons test. Datasets containing a single variable were analyzed using unpaired two‐tailed Student's *t* tests. Data are presented as the mean ± the standard error of the mean (SEM). Data generated from multiple cell lines of the same genotype were pooled, and the number of cell lines and differentiations performed are indicated in figure legends.

## 3 Results

### 3.1 Differentiation of Motor Neurons and Microglia From C9‐HRE iPSCs

**FIGURE 1:**
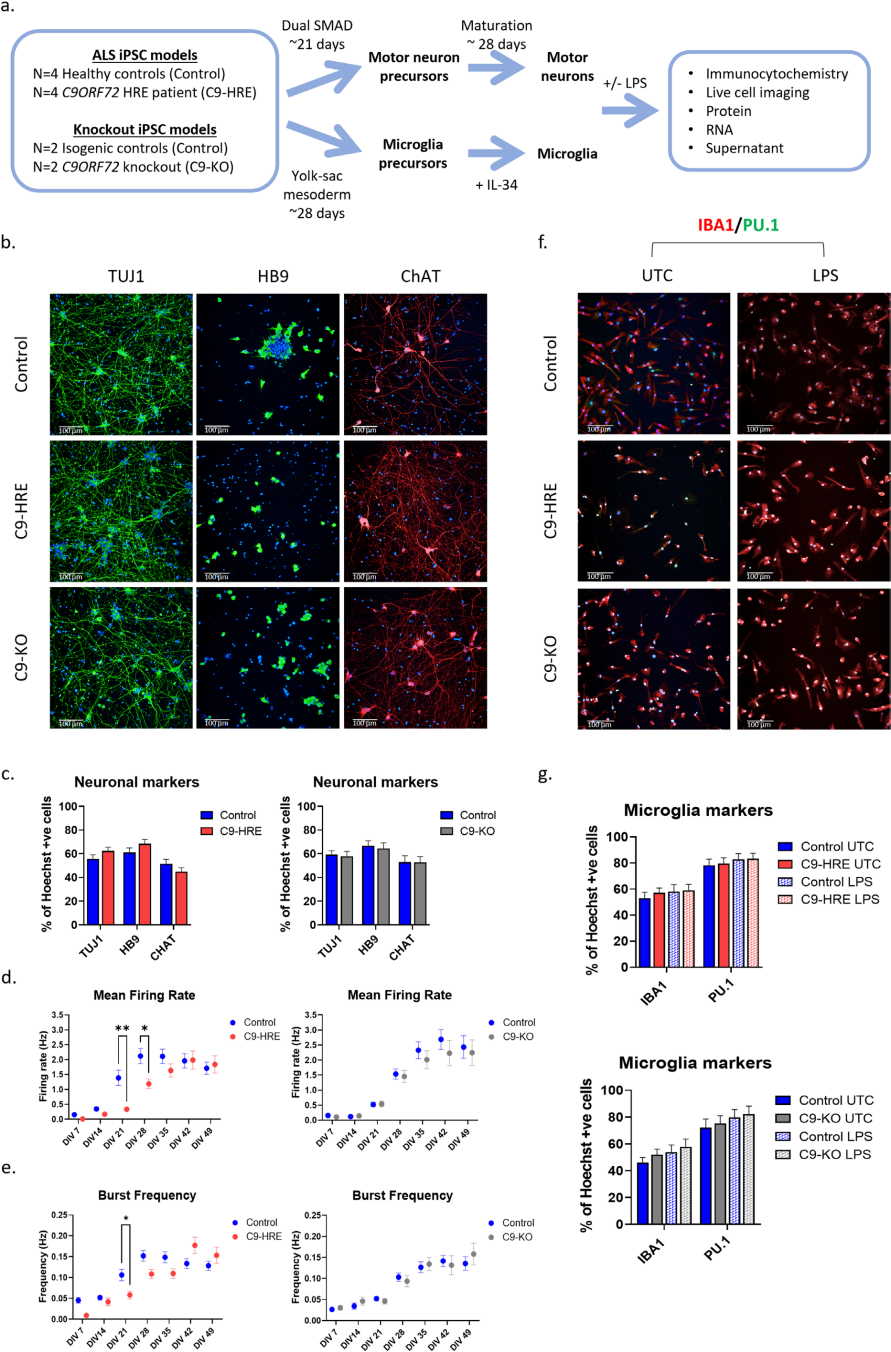
C9‐HRE and C9‐KO iPSCs differentiate to motor neurons and microglia. (a) Schematic of motor neuron and microglia differentiation and analyses performed. (b) Representative immunofluorescence images of iPSC‐derived neurons stained for TUJ1 (green), HB9 (green), and ChAT (red). Scale bar = 100 μm. (c) Quantification of TUJ1, HB9 and ChAT positive cells (*n* ≥ 2 cell lines per genotype, *n* ≥ 2 differentiations per cell line, mean ± SEM). (d) Mean firing rate (Hz) and (e) burst frequency (Hz) of neurons cultured with astrocytes matured over 49 days in vitro (DIV) (*n* ≥ 2 cell lines per genotype, *n* ≥ 2 differentiations per cell line, *n* ≥ 2 wells, mean ± SEM. *p < 0.05, **p < 0.01 in two‐way ANOVA test). (f) Representative immunofluorescence images of iPSC‐derived microglia stained for IBA1 (red) and PU.1 (green). Microglia cultures were untreated (UTC) or LPS stimulated (5 ng/mL 16 h). Scale bar = 100 μm. (g) Quantification of IBA1 and PU.1 positive cells (*n* ≥ 2 cell lines per genotype, *n* ≥ 3 differentiations per cell line, mean ± SEM).

We used well‐established protocols with minor modifications for the differentiation of microglia and motor neurons from iPSCs (Haenseler et al. 2017; Shi et al. 2018). iPSC lines were derived from four independent C9‐HRE ALS patients, and four healthy individuals expected to express *C9ORF72* with nonpathogenic repeat size (< 24) including one isogenic control (Figure 1a, Table S1) (Gijselinck et al. 2016). In addition, we assessed two independent C9‐KO iPSC lines and two isogenic control lines to study potential LOF mechanisms (Figure 1a, Table S1). For this, an isogenic pair of C9‐KO iPSC lines was generated by lentiviral transduction of CRISPR/Cas9 constructs targeting exon 1 of *C9ORF72* in a control iPSC line. The successful generation of a homozygous KO of *C9ORF72* was confirmed by Sanger sequencing and western blot (Figure S1ai,ii). Next, the differentiation efficiency of iPSCs to motor neurons was assessed by immunofluorescence staining, demonstrating that between 40% and 70% of neurons expressed TUJ1, HB9, or ChAT (Figure 1b,c). qRT‐PCR confirmed expression of the genes *MAP2, MNX1*, and *ChAT* compared to undifferentiated iPSCs (Figure S1bi). In addition, we assessed neurite length using TUJ1 immunostaining and found no statistically significant changes in the C9‐HRE or C9‐KO groups compared to controls (Figure S1ci). The impact of C9‐HRE or C9‐KO on the electrical activity of motor neurons was assessed by MEA. Overall, neuronal cultures derived from all cell lines demonstrated spontaneous activity when cocultured with rat astrocytes (Figures 1d,e and S1cii). The mean firing rate and burst frequency increased over 49 days of maturation (DIV 49) and were largely consistent between control and C9‐KO neurons (Figure 1d,e). In C9‐HRE neurons, we found the mean firing rate and burst frequencies to be significantly reduced compared to control at the early timepoints of DIV 21–28 but not after prolonged culture up to DIV 49 (Figure 1d,e).

Next, we assessed the iPSC differentiation of microglia by immunofluorescence staining. In untreated (UTC) microglial cultures, we found a high percentage of cells showing expression of the markers IBA1 (~50%) and PU.1 (~80%) (Figure 1f,g). Similarly, qRT‐PCR analysis showed expression of *ITGAM* compared to undifferentiated iPSCs (Figure S1bii). We found that stimulating the microglial cultures with LPS for 16 h did not alter the differentiation efficiency (Figure 1f,g), but resulted in a small but statistically significant reduction in the cell area of IBA1 positive microglia (Figure S1d), consistent with an activated state. To confirm the functionality of iPSC derived microglia, we assessed phagocytotic activity using pHrodo *E. coli* or Zymosan bioparticles (Figure S1e). Microglia derived from all iPSC lines were capable of phagocytosing bioparticles, and no statistically significant differences were observed between control and C9‐HRE microglia over 6 h of coincubation with bioparticles (Figure S1e). When comparing control and C9‐KO microglia, we found no statistically significant changes using *E. coli* bioparticles but observed a statistically significant reduction in area and intensity of phagocytosed Zymosan bioparticle fluorescence from 3 to 6 h of coincubation (Figure S1e).

Overall, we generated functional motor neurons and microglia from a panel of control, C9‐HRE, and C9‐KO iPSCs. Of note, we identified subtle functional deficits that could aid further investigations in the LOF/GOF mechanisms of *C9ORF72*‐associated disease. Specifically, we identified delayed neuronal firing in C9‐HRE but not C9‐KO motor neurons, and deficient Zymosan bioparticle uptake in C9‐KO, not C9‐HRE, microglia compared to controls.

### 3.2 iPSC‐Derived Motor Neurons and Microglia Demonstrate C9‐HRE Associated Pathology

**FIGURE 2:**
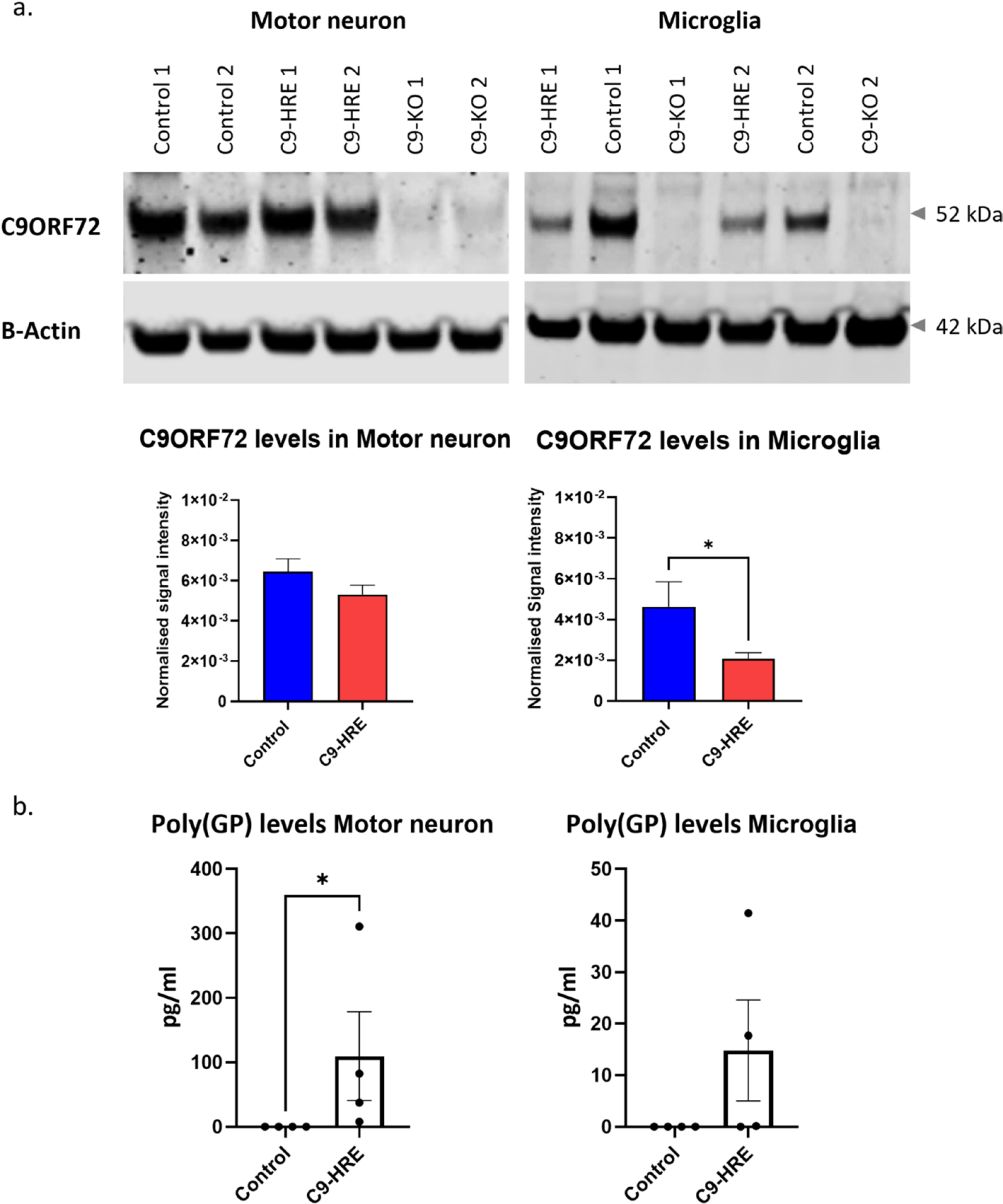
Motor neurons and microglia derived from C9‐HRE iPSCs display C9‐HRE‐associated pathology. (a) Representative western blot for C9ORF72 in iPSC‐derived cultures, and quantification of steady state levels normalized to β‐actin (*n* ≥ 2 cell lines per genotype, *n* ≥ 1 differentiation per cell line, mean ± SEM, *p < 0.05 in two‐tailed unpaired t‐test). (b) Analysis of Poly(GP) levels by MSD assay in iPSC‐derived cultures (*n* = 4 cell lines per genotype represented by dots, *n* = 2 differentiations per cell line, mean ± SEM, *p < 0.05).

We investigated the presence of C9‐HRE‐associated pathological features in iPSC‐derived motor neurons and microglia. First, by qRT‐PCR we found *C9ORF72* expression to be stable between C9‐HRE and control cells (Figure S2a). However, by western blotting analysis we observed a statistically significant decrease (~2 fold) in C9ORF72 protein in C9‐HRE compared to control microglia, but not in C9‐HRE compared to control motor neurons (Figure 2a). Second, we assessed the presence of DPRs by quantifying the levels of Poly(GP) protein using a MSD (Mesoscale Discovery) assay. We detected Poly(GP) in C9‐HRE and not in control microglia or motor neurons (Figure 2b).

Next, we assessed the expression of *TDP‐43* in motor neurons or microglia by qRT‐PCR and western blotting analysis and found no statistically significant differences in C9‐HRE or C9‐KO cultures compared to controls (Figure S2b). To investigate the functionality of TDP‐43, we assessed protein levels within the whole cell and localized to the nucleus or the cytoplasm. Quantification of TDP‐43 in C9‐HRE or C9‐KO motor neuron or microglia cultures showed no statistically significant differences in fluorescence intensity in the whole cell or in the nuclear/cytoplasm ratio compared to controls (Figure S2c).

Overall, we generated functional motor neurons and microglia from iPSCs that demonstrated some C9‐HRE associated pathology, including DPR accumulation and microglia‐specific *C9ORF72* haploinsufficiency.

### 3.3 C9‐HRE Microglia Demonstrate Altered Inflammatory Profile Upon LPS Stimulation

**FIGURE 3:**
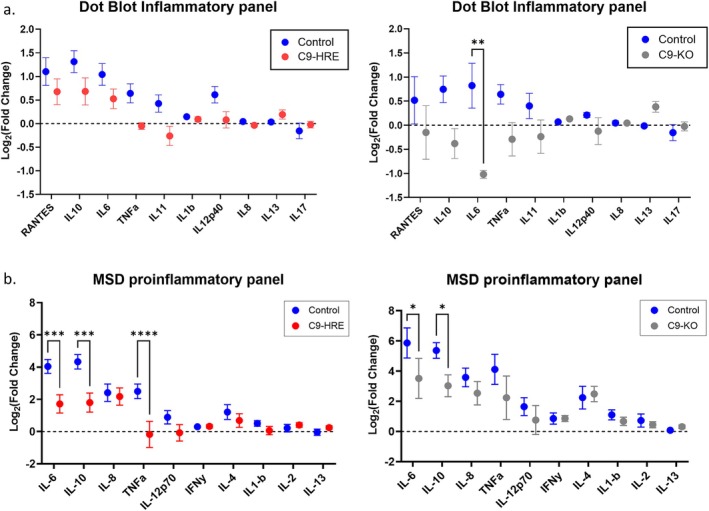
Microglia derived from C9‐HRE and C9‐KO iPSCs demonstrate dysregulated cytokine secretion. Assessment of inflammatory cytokines secreted from LPS‐stimulated (5 ng/mL for 16 h) microglia cultures assessed by (a) dot blot and (b) MSD assay (*n* ≥ 2 cell lines per genotype, *n* ≥ 1 differentiation per cell line, mean log_2_ LPS/UTC fold change ± SEM, *p < 0.05, **p < 0.01, ***p < 0.001, ****p < 0.0001 in two‐way ANOVA test).

To assess the inflammatory profile of iPSC‐derived microglia, we measured the levels of cytokines in cell culture media with and without LPS stimulation. The concentration of LPS was determined by ELISA, using the known inflammatory marker IL‐6 (Figure S3a). Initially, to profile the broad inflammatory response, a panel of 40 inflammatory markers was measured by dot blot analysis. Control microglia were reactive to LPS treatment and demonstrated marked upregulation of RANTES, IL‐10, IL‐6, TNF‐α, IL‐11, and IL‐12p40. In comparison, the fold changes in the secretion of these markers upon LPS stimulation were reduced in C9‐HRE and C9‐KO microglia (Figure 3a). We did not observe notable changes in other markers assessed (Figure S3b). Next, we performed an MSD assay to measure the secreted levels of a panel of 10 inflammatory cytokines (Figure 3b). Comparable to dot blot results, we also observed reductions in the secretion of IL‐6, IL‐10, and TNF‐α in LPS‐stimulated C9‐HRE and C9‐KO compared to control microglia (Figure 3b). We did not observe notable changes in the secreted levels of other markers in the panel.

**FIGURE 4:**
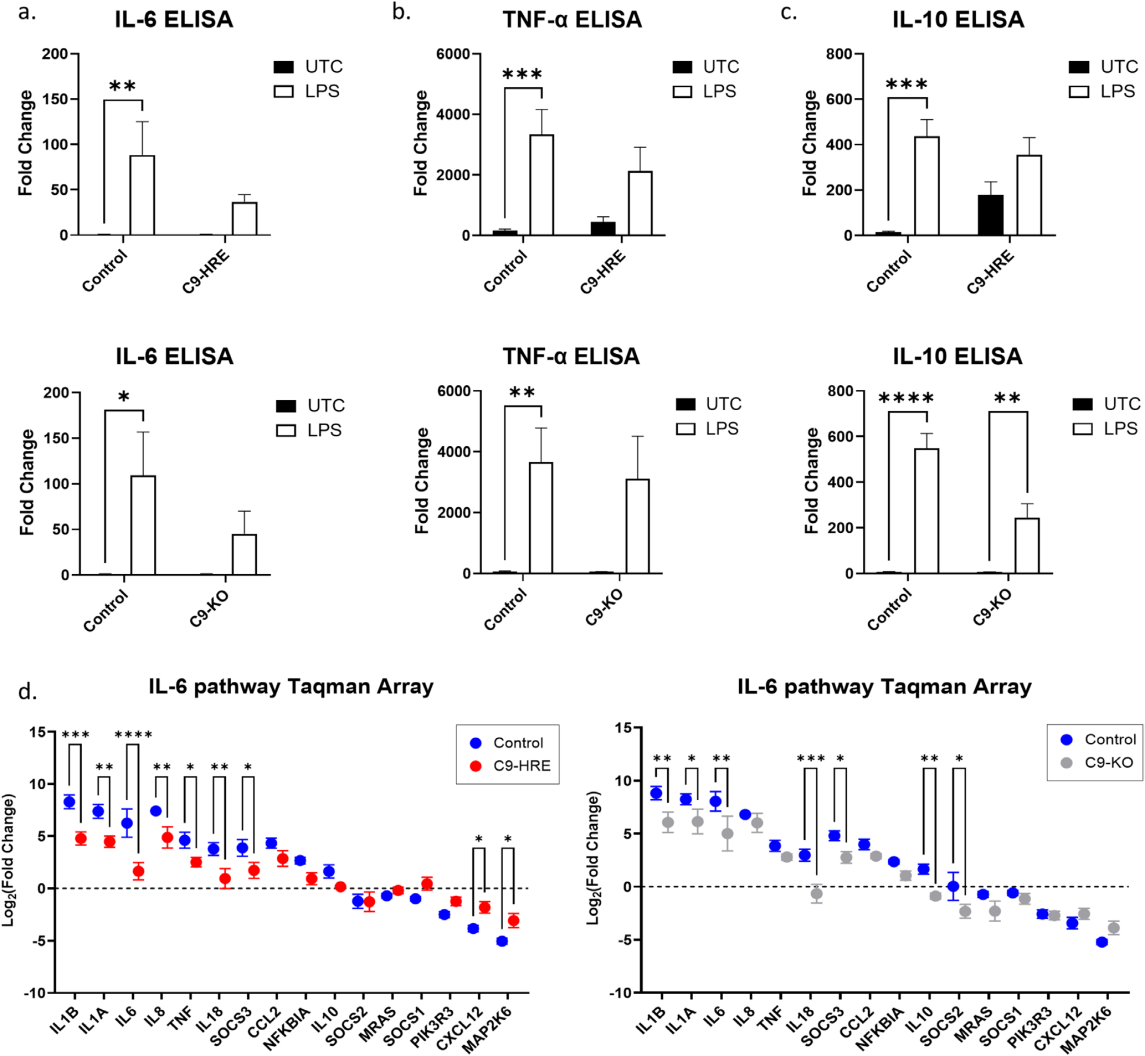
Microglia derived from C9‐HRE and C9‐KO iPSCs demonstrate reduced IL‐6, TNF‐α and IL‐10 secretion and expression. Assessment of (a) IL‐6, (b) TNF‐α, and (c) IL‐10 secretion from LPS‐stimulated (5 ng/mL for 16 h) microglia by ELISA (*n* ≥ 2 cell lines per genotype, *n* ≥ 1 differentiation per cell line, mean LPS/UTC fold change ± SEM, *p < 0.05, **p < 0.01, ***p < 0.001, ****p < 0.0001 in two‐way ANOVA or unpaired *t* test). (d) Expression of top 16 differential genes in LPS‐stimulated (5 ng/mL for 16 h) microglia measured by qRT‐PCR (*n* = 2 cell lines per genotype, *n* ≥ 2 differentiations per cell line, mean log_2_ LPS/UTC fold change ± SEM, *p < 0.05, **p < 0.01, ***p < 0.001, ****p < 0.0001 in two‐way ANOVA test).

To further validate our findings, we performed individual ELISAs on the secreted levels of IL‐6, IL‐10, and TNF‐α (Figure 4a–c). As expected, in control microglia, we observed statistically significant upregulation in the secretion of IL‐6, TNF‐α and IL‐10 with LPS stimulation (Figure 4a–c). In C9‐HRE and C9‐KO microglia, we observed an increase in these secretory markers with LPS stimulation; however, the fold change was reduced compared to control microglia and was not statistically significant (Figure 4a–c). Interestingly, C9‐HRE and C9‐KO microglia primed with alternative stimuli, such as TNF‐α and IFN‐γ, also showed reduced IL‐6 secretion by ELISA, suggesting that the impairment extends beyond Toll‐like receptor 4 (TLR4) signaling (Figure S3a).

Finally, as we observed robust alterations in IL‐6 secretion from C9‐HRE and C9‐KO compared to control microglia across multiple assays, we sought to assess whether these changes could also be observed at the transcript level using a Taqman Array panel (Figures 4d and Figure S4). Out of 92 genes assessed, we found that *IL‐1B, IL‐1A, IL‐6, IL‐18*, and *SOCS3* were downregulated in C9‐HRE or C9‐KO microglia compared to controls. In addition, *IL‐8* and *TNF‐α* were significantly downregulated only in C9‐HRE microglia, and *IL‐10* and *SOCS2* were significantly downregulated only in C9‐KO microglia compared to controls (Figures 4d and S4). Interestingly, *CXCL12* and *MAP2K6* were significantly upregulated in C9‐HRE microglia compared to controls (Figures 4d and S4).

Overall, C9‐HRE and C9‐KO microglia demonstrated reduced expression and secretion of inflammatory markers, particularly IL‐6, compared to controls. These results, along with the presence of C9ORF72 haploinsufficiency in C9‐HRE microglia, indicate that the changes in C9‐HRE microglia inflammatory profile are potentially linked to LOF mechanisms associated with the C9‐HRE mutation.

### 3.4 Establishment and Assessment of Motor Neuron and Microglia Cocultures From C9‐HRE iPSCs

**FIGURE 5:**
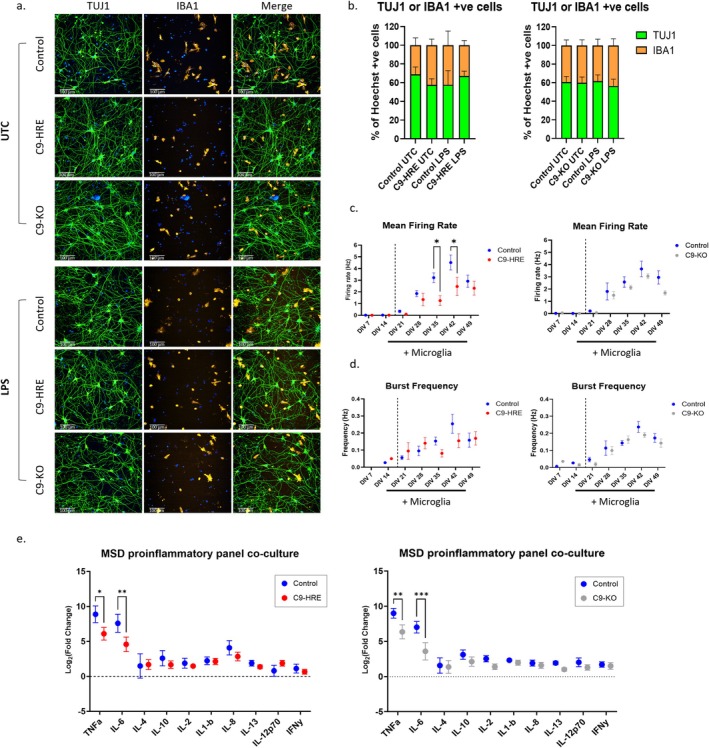
Motor neuron/microglia cocultures derived from C9‐HRE and C9‐KO iPSCs recapitulates monoculture phenotypes. (a) Representative immunofluorescence images of iPSC derived neuron/microglia cocultures with or without LPS stimulation (5 ng/mL for 16 h), stained for TUJ1 (green) and IBA1 (orange). Scale bar = 100 μm. (b) Quantification of TUJ1 (green) and IBA1 (red) positive cells (*n* = 2 cell lines per genotype, *n* = 5 differentiations per cell line, mean ± SEM). (c) Mean firing rate (Hz) and (d) burst frequency (Hz) of neuron/microglia cocultures with astrocytes assessed by MEA (*n* = 2 cell lines per genotype, *n* = 2 differentiations per cell line, mean ± SEM. *p < 0.05 in two‐way ANOVA test). (e) Assessment of inflammatory cytokines secreted from LPS‐stimulated (5 ng/mL 16 h) or UTC neuron/microglia cocultures by MSD assay (*n* ≥ 2 cell lines per genotype, *n* ≥ 1 differentiations per cell line, mean log_2_ LPS/UTC fold change ± SEM, *p < 0.05, **p < 0.01, ***p < 0.001 in two‐way ANOVA test).

To investigate the potential impact of the altered inflammatory profile of C9‐HRE microglia on motor neuron health and function, we established motor neuron and microglia cocultures. To validate the coculture model, we performed immunofluorescence staining and showed that ~50% of cells stained for either the neuronal marker TUJ1 or the microglial marker IBA1 (Figure 5a,b). We did not observe statistically significant alterations in the number of neurons in C9‐HRE or C9‐KO cocultures compared to controls, or when cocultures were stimulated with LPS (Figure 5a,b). In addition, we assessed neurite length in cocultures using the TUJ1 neuronal marker and found no statistically significant changes in the C9‐HRE or C9‐KO neurons compared to controls, or when cocultures were stimulated with LPS (Figure S5i). To assess the electrical competency of motor neurons in cocultures, rat astrocytes were added to the cocultures. The addition of microglia after DIV 14 did not alter the ability of motor neurons to initiate spontaneous firing (Figures 5c,d and S5ii). Furthermore, the mean firing rate and burst frequency increased up to DIV 35 and were largely consistent between C9‐KO and control neurons. However, we observed a significant reduction in firing rate in C9‐HRE motor neurons compared to control at DIV 35–42 (Figure 5c).

Finally, to determine if reduced cytokine secretion observed in C9‐HRE and C9‐KO microglia upon LPS stimulation was maintained in coculture with neurons, the secreted levels of 10 cytokines from microglia in coculture were assessed using the MSD assay (Figure 5e). We observed reductions in the secretion of TNF‐α and IL‐6 upon LPS stimulation of C9‐HRE and C9‐KO compared to control microglia in coculture (Figure 5e). Interestingly, we did not observe significant changes in secreted IL‐10 upon LPS stimulation, as was observed in C9‐HRE and C9‐KO microglia monocultures (Figure 5e). No changes in the secreted levels of other inflammatory markers were detected in the tested panel.

Overall, we generated motor neuron and microglia cocultures and found no statistically significant changes in the number or function of motor neurons, apart from a reduction in firing rate in C9‐HRE motor neurons. However, C9‐HRE or C9‐KO microglia in cocultures showed reduced levels of secretory cytokines upon LPS stimulation compared to controls, similar to microglia in monoculture. The potential impact of the dysregulated inflammatory response in C9‐HRE or C9‐KO microglia on motor neurons remains to be determined.

### 3.5 Single‐Cell Transcriptomic Profiling of C9‐HRE Motor Neuron and Microglia Cocultures

**FIGURE 6:**
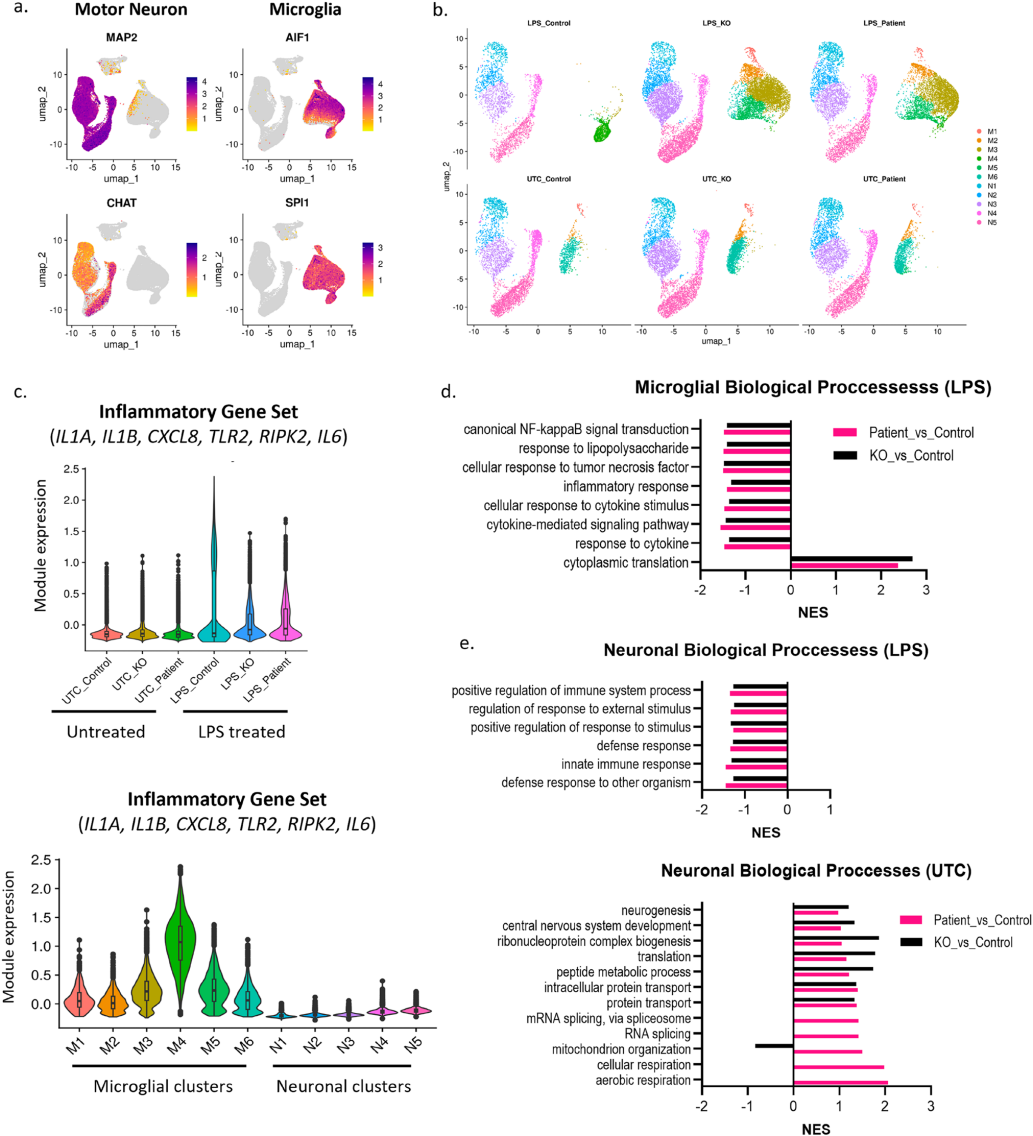
Motor neuron/microglia cocultures derived from C9‐HRE and C9‐KO iPSCs demonstrate altered inflammatory processes. UMAP visualization (a) of relative expressions of *MAP2, ChAT, AIF1* (encoding IBA1), and *SPI1* to identify neuronal or microglia cell clusters and (b) of all cell clusters identified in motor neuron/microglia cocultures (log_2_ UMI count). (c) Violin plots of the altered expression of an inflammatory gene set (*IL‐1A, IL‐1B, CXCL8, TLR2, RIPK2,* and *IL‐6*) across samples or cell clusters. Top significantly altered biological processes in (d) LPS stimulated (5 ng/mL for 16 h) microglial cell group and (e) motor neurons cell groups, identified through gene set enrichment analysis (GSEA). Normalized enrichment score (NES) reflects the direction and statistical significance of change.

To further elucidate the potential interplay between motor neurons and microglia in C9‐HRE‐associated ALS, we performed scRNAseq on motor neuron and microglia cocultures. We sequenced isogenic control, C9‐HRE (patient) and C9‐KO (knockout) cocultures that were either untreated (UTC) or stimulated with LPS. We segregated microglia and motor neuron populations using cell type specific marker genes (Figures 6a and S6a), assessed differentially expressed genes (DEGs) and performed GSEA to identify mechanisms associated with C9‐HRE mediated ALS pathology.

In microglia identifying cell clusters, we observed minimal changes without LPS stimulation across genotypes. However, in LPS‐treated control cocultures, a distinct microglial cell cluster (M4) can be observed compared to LPS‐treated C9‐HRE and C9‐KO cocultures (Figure 6b). Notably, within the M4 microglial cell cluster, we found the highest expression of a set of genes known to be associated with the immune activation process, including *IL‐1A, IL‐1B, CXCL8, TLR2*, and *RIPK2* (Figure 6c). Consequentially, we observed downregulations in immune activation related biological processes in C9‐HRE and C9‐KO LPS stimulated microglia, including canonical NF‐κB signal transduction (GO:0007249), inflammatory response (GO:0006954), and response to cytokine (GO:0034097) (Figures 6d and S6bi). This result supports our cytokine secretion assays, where a reduction in inflammatory response was also observed in C9‐HRE and C9‐KO microglia with LPS stimulation compared to controls. In addition, we also found ribosomal proteins to be largely upregulated in LPS stimulated C9‐HRE and C9‐KO microglia compared to control, resulting in a significant upregulation in cytoplasmic translation (GO:0002181) (Figures 6d and S6bi).

In neuron identifying cell clusters, a reduction in immune (GO:0006955) and inflammatory (GO:0006954) processes was also observed in C9‐HRE and C9‐KO compared to controls with LPS stimulation (Figures 6e and S6biii). In UTC C9‐HRE and C9‐KO neurons, biological processes involved in protein transport (GO:0015031) and neurogenesis (GO:0022008) were altered compared to controls (Figures 6e and S6bii). Interestingly, we identified potential GOF‐associated mechanisms exclusively altered in UTC C9‐HRE and not C9‐KO neurons compared to controls, including genes involved in aerobic respiration (GO:0009060) and RNA splicing (GO:0008380) (Figures 6e and S6bii).

## 4 Discussion

The role of microglia and inflammation has been a major focus in ALS research (Clarke and Patani 2020). Studies to date have not clearly delineated the specific contributions of motor neurons and microglia in ALS pathology. In this study, we generated motor neuron and microglia cocultures from C9‐HRE and C9‐KO iPSCs to investigate ALS‐associated phenotypes that manifest in either or both cell types. Primarily, we observed dysregulations in the expression and secretion of inflammatory cytokines from LPS‐stimulated C9‐HRE and C9‐KO iPSC‐derived microglia. This finding, along with C9ORF72 haploinsufficiency in C9‐HRE microglia, suggests that an altered inflammatory process is potentially caused by the *C9ORF72* LOF. We report that the secretion of pro‐inflammatory factors such as IL‐6 and TNF‐α is reduced in LPS‐stimulated C9‐HRE and C9‐KO microglia compared to controls. Although this finding does not fully recapitulate previous studies utilizing iPSC‐derived C9‐HRE microglia, where no functional change or an increased pro‐inflammatory profile was observed (Banerjee et al. 2023; Lorenzini et al. 2023; Vahsen et al. 2023), our results support the role of *C9ORF72* in the regulation of immune responses. The role of *C9ORF72* in microglial immune function has been reported by studies performed on *C9ORF72* null mice, where microglia demonstrate altered lysosomal trafficking and immune response, although neuronal viability was not impacted (O'Rourke et al. 2016; Sudria‐Lopez et al. 2016). While excessive microglial activation is commonly associated with neurodegeneration, an insufficient or dysregulated immune response may likewise impair neuronal support, synaptic maintenance, and debris clearance (Gao et al. 2023), thereby contributing to *C9ORF72*‐related ALS/FTD pathology.

We observed no overt changes in C9‐HRE or C9‐KO iPSC‐derived motor neurons in monoculture or coculture with microglia. Similarly, several reports of C9‐HRE iPSC‐derived motor neuron models did not demonstrate major changes such as neuronal loss; however, extensive phenotypic studies identified alterations in synaptic activity, neurotransmitter release, calcium homeostasis, increased endoplasmic reticulum (ER) stress, and susceptibility to apoptosis (Abo‐Rady et al. 2020; Bursch et al. 2019; Dafinca et al. 2020, 2016; Devlin et al. 2015). In one study, comparison to C9‐KO iPSC‐derived motor neurons did not recapitulate reduced axonal trafficking observed with C9‐HRE motor neurons compared to isogenic controls, suggesting this alteration could be linked to C9‐HRE GOF mechanisms (Abo‐Rady et al. 2020). However, in aged C9‐KO mice, microglia‐dependent synaptic pruning can be reduced and lead to defects in learning and memory (Lall et al. 2021). Thus, further investigation into the broader impact of C9ORF72 deficient microglia on neuronal function beyond neuroinflammation is warranted. Furthermore, in mouse models, C9‐KO or C9‐HRE and the presence of RNA foci and DPRs have been shown to be insufficient to cause motor neuron degeneration (Koppers et al. 2015; O'Rourke et al. 2015), although loss of *C9ORF72* can lead to synaptic dysfunction and excitotoxicity (Xiao et al. 2019). Finally, previous studies have demonstrated that the development of autophagy deficits in C9‐HRE microglia can increase the vulnerability of C9‐HRE motor neurons to excitotoxicity (Banerjee et al. 2023), and LPS primed and pro‐inflammatory C9‐HRE microglia can increase the expression of apoptotic markers in healthy motor neurons (Vahsen et al. 2023). Taken together, it is possible that increasingly complex and aged models are required for motor neurons to develop a clear degenerative phenotype in vitro. In a C9‐HRE iPSC‐derived cerebral organoid slice model aged up to DIV 240 and recapitulating a mature cortical architecture, disturbances were observed in neurons and astrocytes relating to autophagy and DNA repair (Szebenyi et al. 2021). However, the authors noted the absence of microglia and vasculature in the organoid model, which could attribute to the lack of marked neurodegeneration (Szebenyi et al. 2021).

In this study, scRNAseq of LPS stimulated C9‐HRE and C9‐KO cocultures revealed dysregulations in biological processes associated with the function(s) of *C9ORF72*. In LPS primed C9‐HRE and C9‐KO microglia, we identified the loss of a LPS responsive microglia subpopulation, correlating with reductions in cytokine‐mediated signaling and inflammatory response pathways. In UTC C9‐HRE motor neurons, we identified DEGs associated with mitochondrial, protein transport, and RNA splicing processes, all of which are key mechanisms involved in ALS pathology (Mead et al. 2023). This supports the notion that transcriptomic deviations can manifest in neurons prior to the manifestation of robust pathological changes within a prodromal disease phase. The above‐mentioned pathways are also known to be associated with *C9ORF72* function. For instance, *C9ORF72* can participate in protein transport by functioning in a complex as a guanine nucleotide exchange factor (GEF) for RAB GTPases to regulate the autophagy‐lysosomal pathway (Corbier and Sellier 2017; Sellier et al. 2016; Sullivan et al. 2016; Yang et al. 2016). Accordingly, decreased or loss of *C9ORF72* in neuron cultures has been shown to impair endosome maturation (Beckers et al. 2023) and lead to autophagy deficits (Corbier and Sellier 2017; Webster et al. 2016). Furthermore, RNA dysregulation is a key contributor to ALS pathology, and multiple ALS associated genes such as *SOD1*, *TARDPB* (encoding TDP‐43), and *C9ORF72* can participate in RNA metabolism including RNA transcription, RNA splicing, RNA transport, and RNA stabilization (Butti and Patten 2018). In C9‐HRE‐associated ALS, the presence of RNA foci and DPRs can elicit RNA mediated toxicity (McEachin et al. 2020) or impair nuclear import of transcripts by interacting with the nucleocytoplasmic transport protein Ran GTPase‐activating protein (RanGAP) in C9‐HRE iPSC derived neurons (Zhang et al. 2015).

In summary, we have established motor neuron and microglia cocultures to investigate the role of neuroinflammation and the interplay between the two cell types in ALS pathology. Overall, we provided evidence to support the role of microglia‐mediated inflammation in ALS and identified changes in immune responses that could potentially be indicative of pre‐onset disease. Our coculture model captured transcriptomic alterations in key pathways of ALS pathology, offering it as a valuable platform for mechanistic and proteomic studies to better understand ALS biology and as a tool in the development of novel ALS therapeutics.

## Author Contributions

Y.G. contributed to study conception and design, data collection, analysis and interpretation of results, and manuscript preparation. J.L.B. and H.S. contributed to study design, data analysis, and manuscript review. G.A.O., C.F.B., J.M.M., N.G.W., E.D.D., B.G., and D.M.T. contributed to study conception and design, interpretation of results, and manuscript review.

## Conflicts of Interest

All authors were or are employees of Astex Pharmaceuticals.

## Supporting information


Figure S1‐S6.

## Data Availability

The data that support the findings of this study are available from the corresponding author upon reasonable request. RNA sequencing data have been submitted to the EMBL database under ArrayExpress accession E‐MTAB‐14891.
