# Supplementary material for: Altered Inflammatory Signature in a C9ORF72 ‐ALS iPSC‐Derived Motor Neuron and Microglia Coculture Model
Source: Glia. 2025 Sep 15;74(1):e70084. doi: 10.1002/glia.70084 (PMC12667000; doi:10.1002/glia.70084)
Supplement: Supplementary file 1 — Figure S1‐S6. [file GLIA-74-0-s001.pdf]

iPSC line	Primary tissue	Sex	Age at Tissue Sampling	Gene mutation	Parental cell line
CS29iALS-C9n1	Fibroblast	Male	47	C9orf72 HRE	N/A
CS52iALS-C9n6	Fibroblast	Male	49	C9orf72 HRE	N/A
UCLi001-A	Fibroblast	Male	50-54	C9orf72 HRE	N/A
UCLi004-A	Fibroblast	Male	50-54	C9orf72 HRE	N/A
CS29iALS-C9n1.ISOT2RB4	Fibroblast	Male	47	Control	CS29iALS-C9n1
RBi001-A	Fibroblast	Male	45-49	Control	N/A
WTSIi081-A	Fibroblast	Male	50-54	Control	N/A
WTSIi081-B	Fibroblast	Male	50-54	Control	N/A
CS83iCTR-33n1	Fibroblast	Female	21	Control	N/A
CS83iCTR-n1.C9KOHomoD2	Fibroblast	Female	21	C9orf72 KO Homo	CS83iCTR-33n1
CS29iALS-C9n1.ISOT2RB4 C3.6	Fibroblast	Male	47	C9orf72 KO Homo	CS29iALS-C9n1.ISOT2RB4

Table S1 Summary of iPSC lines

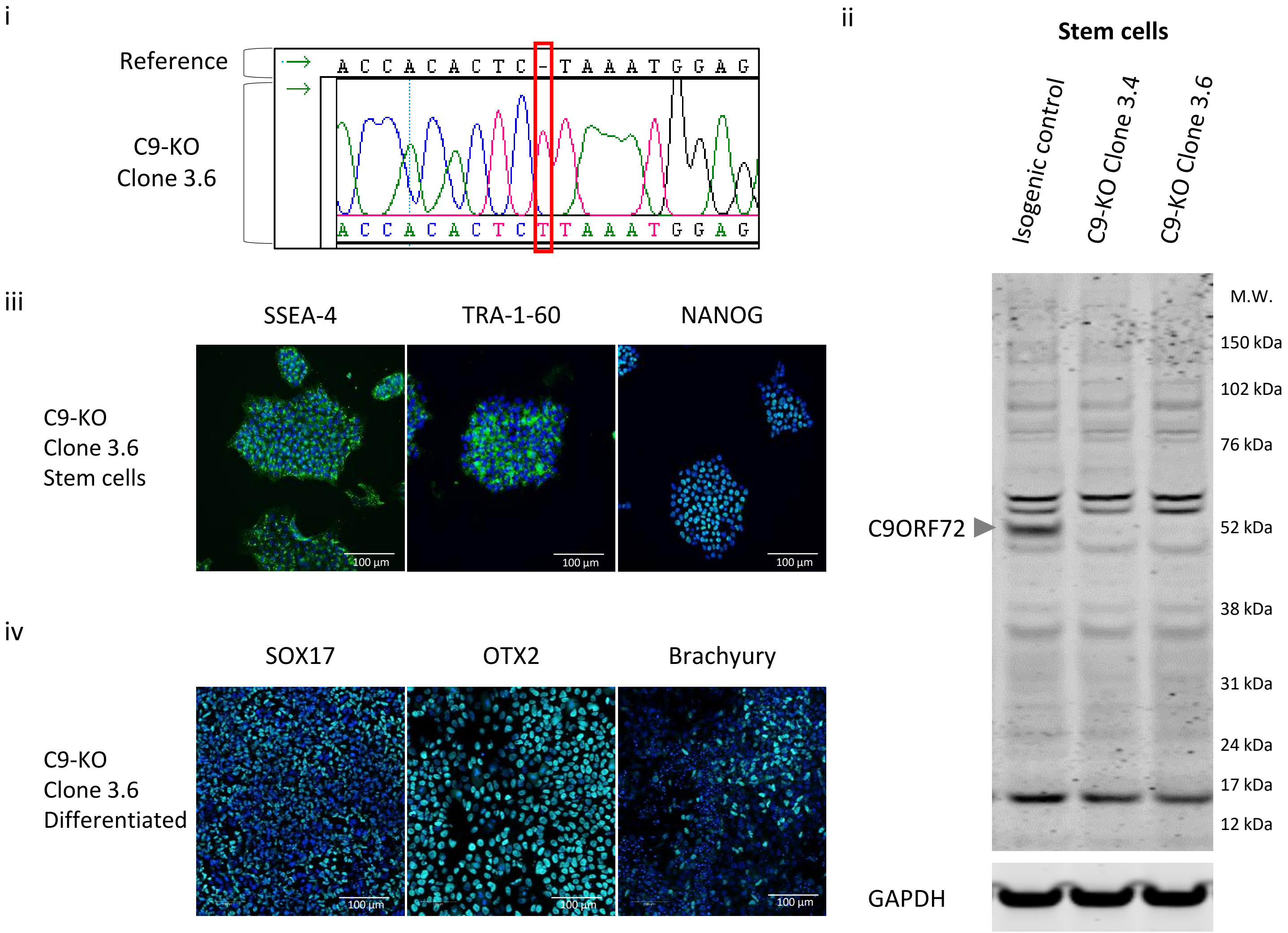

Figure S1a Validation of iPSC line with *C9ORF72* CRISPR/Cas9 knockout generated from CS29iALS-C9n1.ISOT2RB4. (i) Sanger sequencing confirming homozygous point mutation in *C9ORF72* C9-KO clone 3.6 iPSCs (NM_001256054.3:c.200dup). The insertion leads to a frameshift and premature stop codon. (ii) Western blot analysis confirming loss of C9ORF72 in C9-KO clones. (iii) Representative immunofluorescence images of C9-KO clone 3.6 iPSCs stained for pluripotency markers SSEA-4, TRA-1-60 and NANOG (green). Scale bar = 100 µm. (iv) Representative immunofluorescence images of C9-KO clone 3.6 differentiated cultures, stained for endoderm (SOX17), mesoderm (Brachyury) and ectoderm (OTX2) markers. Scale bar = 100 µm.

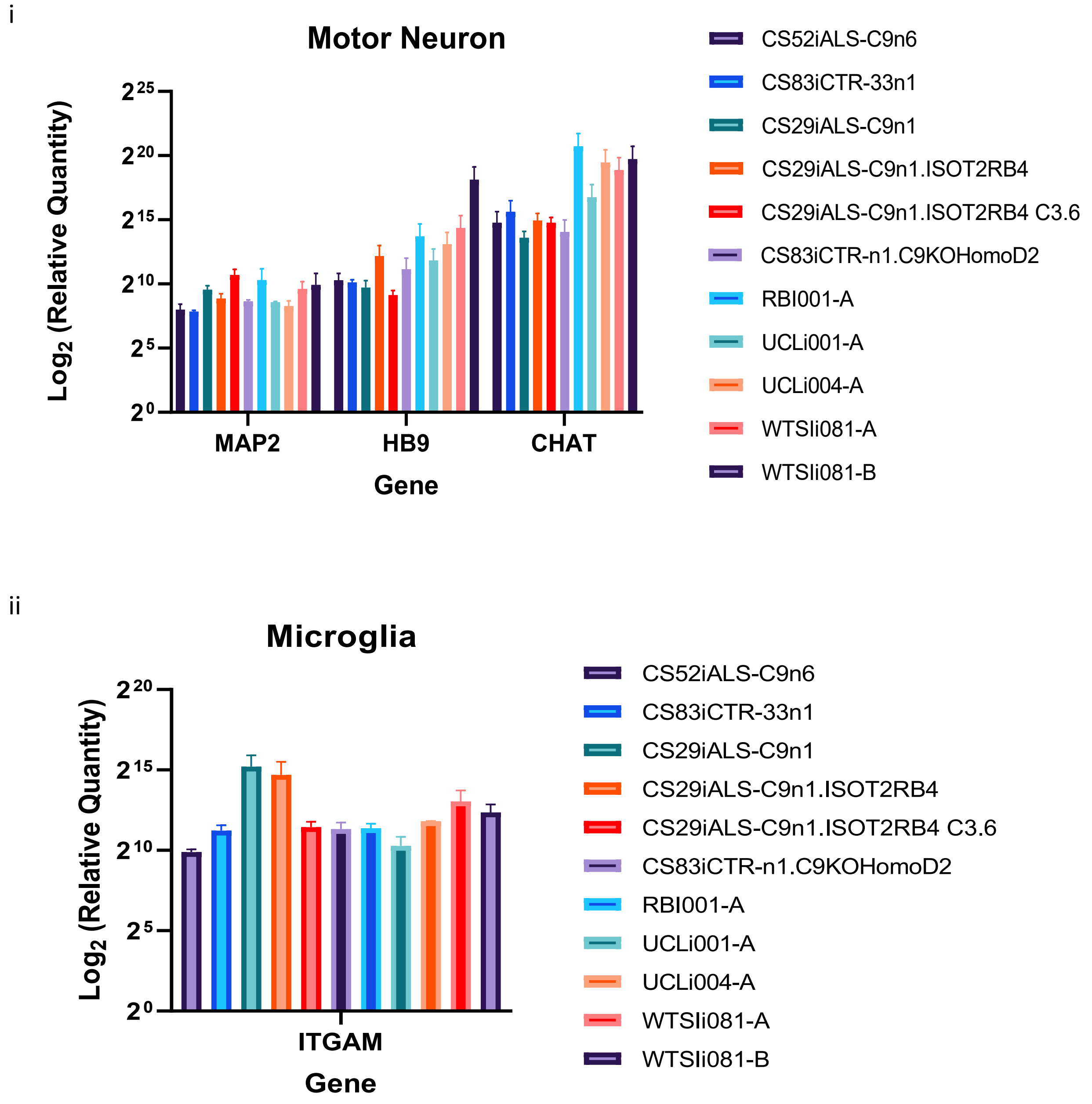

Figure S1b Assessment of neuron and microglia markers in iPSC derived cultures by qRT-PCR .
Relative expressions of motor (i) neuron markers *MAP2, MNX1, ChAT* and (ii) microglia marker *ITGAM* in iPSC derived cultures, normalised to levels in undifferentiated iPSCs (n≥1 differentiations per cell line, mean $\log_2$ relative quantification ($2^{-\Delta\Delta CT}$) ± S.E.M.).

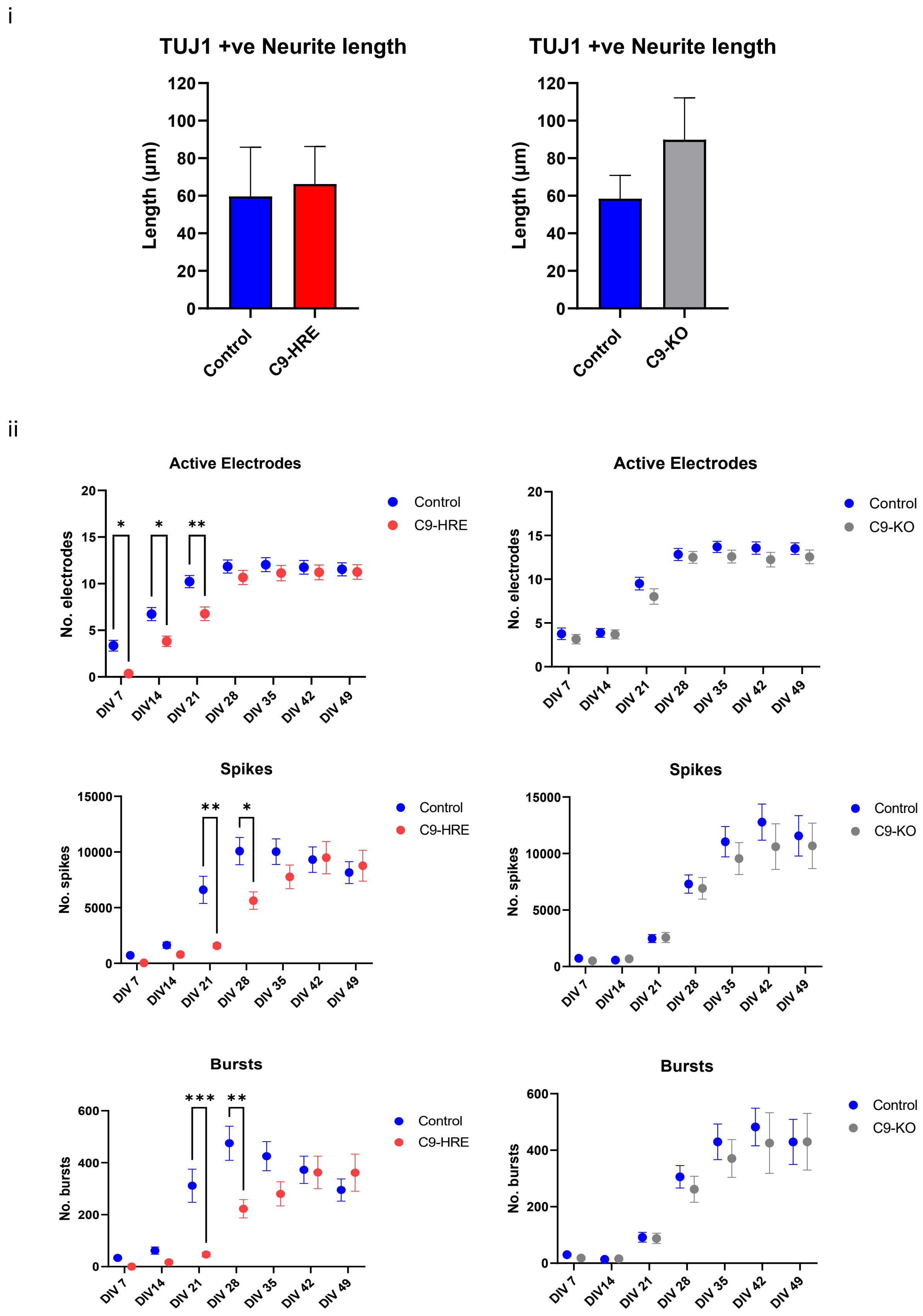

Figure S1c Assessment of neurite outgrowth and electrical activity in iPSC derived neurons.
(i) Neurite lengths of TUJ1 immunostained iPSC derived neurons (n≥2 cell lines per genotype, n≥2 differentiations per cell line, mean ± S.E.M.). (ii) The number of active electrode, spikes and bursts detected from iPSC derived neurons cultured with astrocytes by MEA (n≥2 cell lines per genotype, n≥2 differentiations per cell line, mean ± S.E.M. *p<0.05, **p<0.01, ***p<0.001 in two-way ANOVA test).

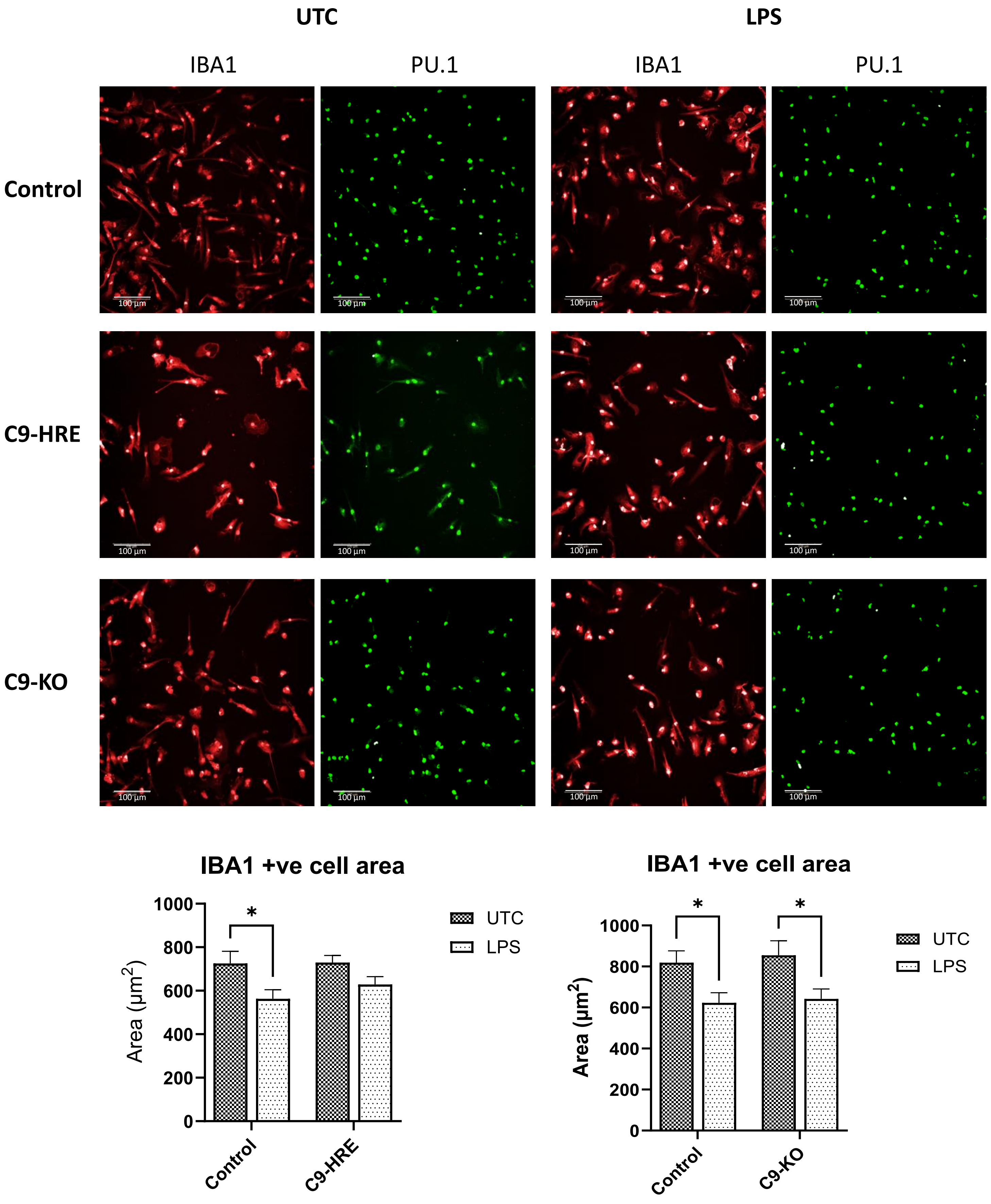

Figure S1d Assessment of cell area of iPSC derived microglia.
Representative immunofluorescence images of iPSC derived microglia stained for IBA1 and PU.1, and quantification of cell area (n≥2 cell lines per genotype, n≥3 differentiations per cell line, mean ± S.E.M. *$p<0.05$ in two-way ANOVA test). Scale bar = 100 µm.

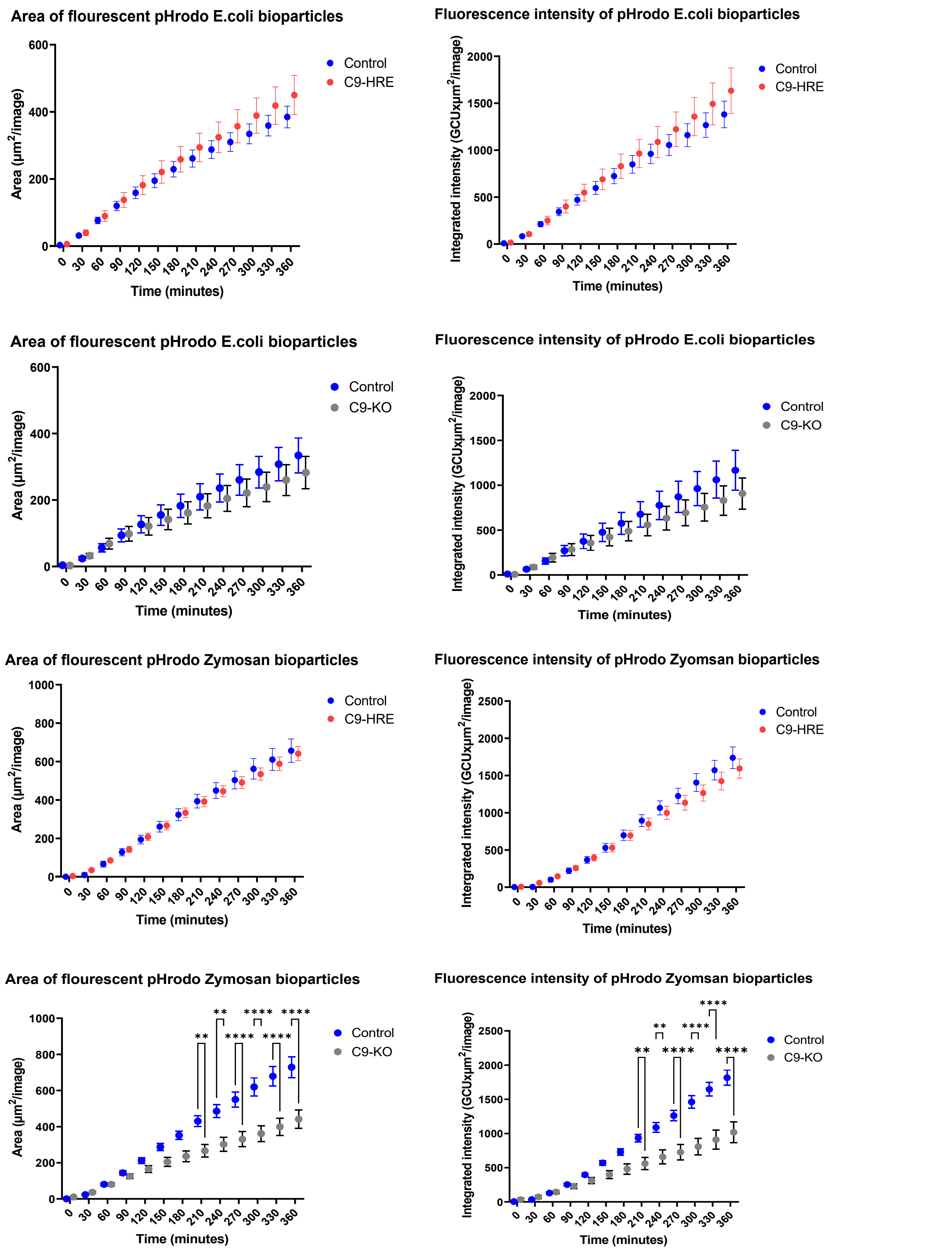

Figure S1e Assessment of phagocytotic activity in iPSC derived microglia.
Quantification of the area (GCUxµm² per image) and fluorescence intensity (GCUxµm² per image) of pHrodo™ E.coli and Zymosan bioparticles internalised by iPSC derived microglia over 6 hours (n≥2 cell lines per genotype, n≥1 differentiation per cell line, mean ± S.E.M. **p<0.01, ****p<0.0001 in two-way ANOVA test).

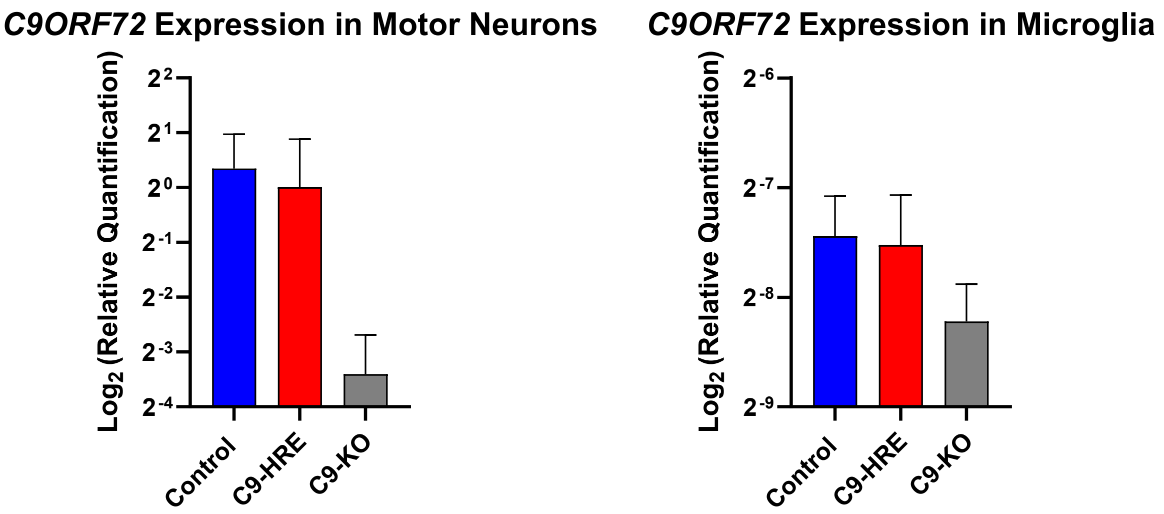

Figure S2a Assessment of *C9ORF72* transcript levels in iPSC derived neuron and microglia cultures.
(n=4 cell lines per genotype, n≥2 differentiations per cell line, mean $\log_2$ relative quantification ($2^{-\Delta CT}$) ± S.E.M.).

i

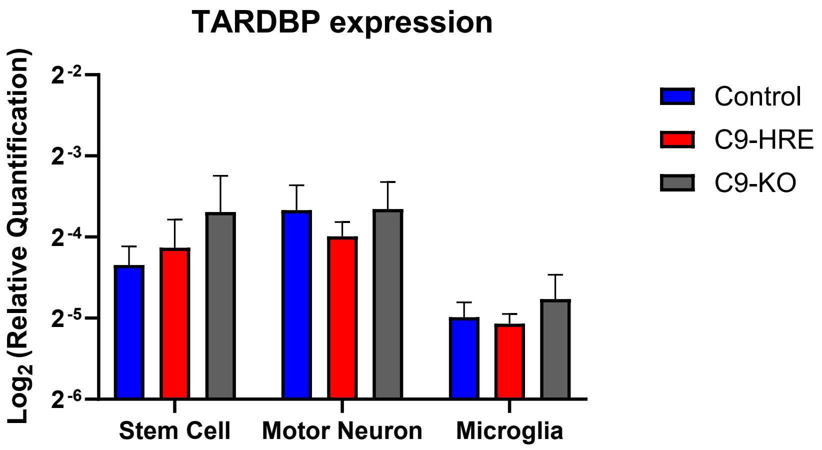

ii

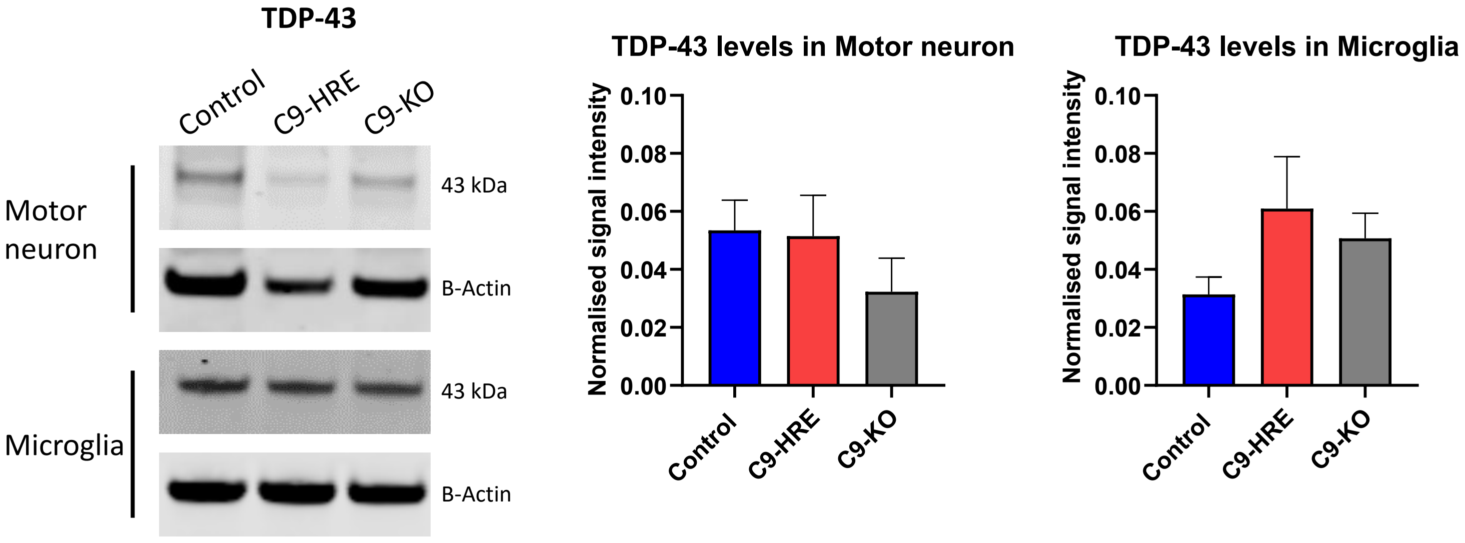

Figure S2b Assessment TDP-43 expression and steady state levels.
(i) *TARDBP* levels in iPSC derived neuron and microglia cultures measured by qRT-PCR (n≥2 cell lines per genotype, n=2 differentiations per cell line, mean $\log_2$ relative quantification ($2^{-\Delta CT}$) ± S.E.M.). (ii) Representative western blots and quantification for TDP-43 in iPSC derived neuron or microglia cultures (n≥2 cell lines per genotype, n=2 differentiation per cell line, mean ± S.E.M.).

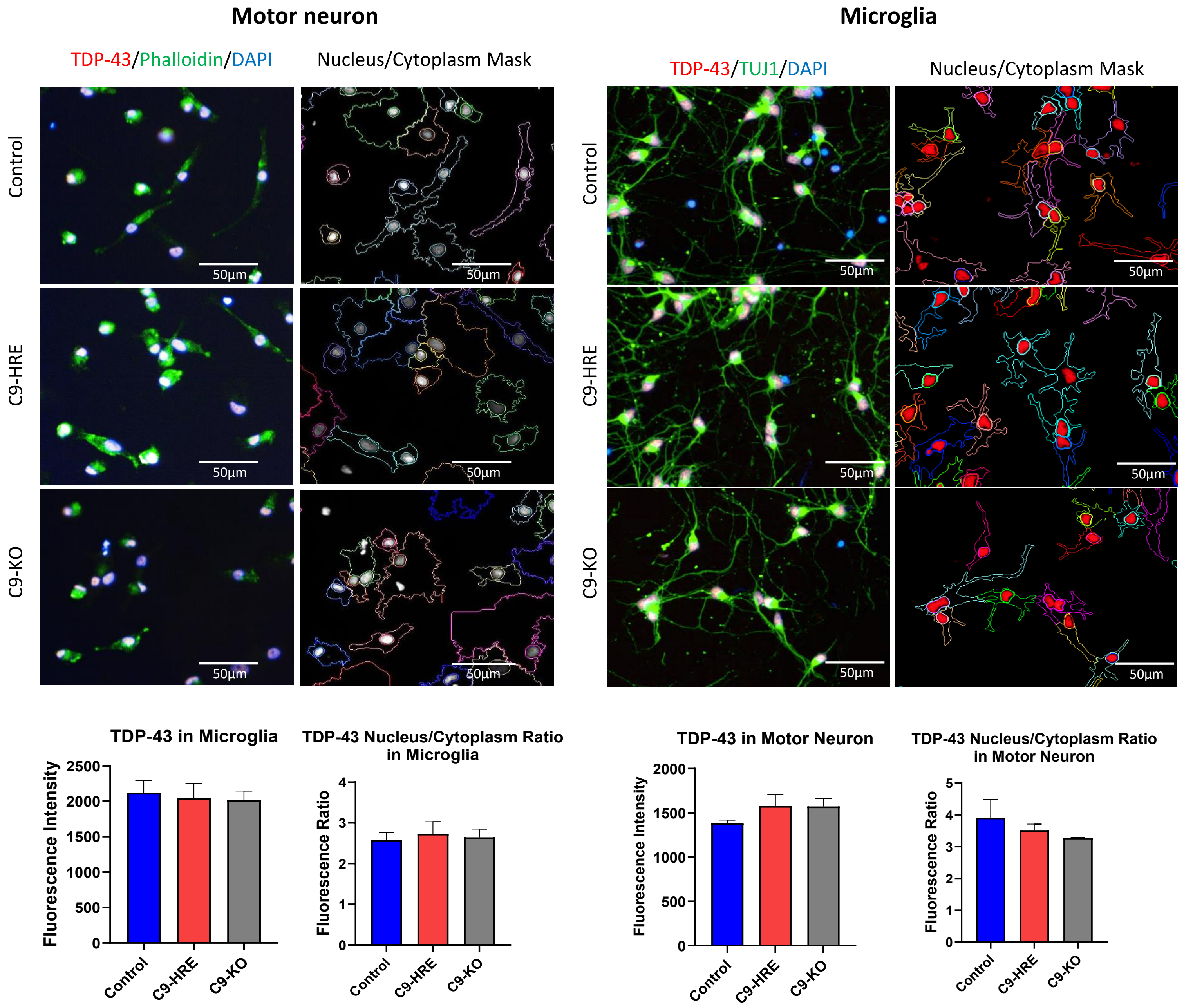

Figure S2c Assessment of TDP-43 localisation.
Representative immunofluorescence images and quantification of TDP-43 (red) and Phalloidin or TUJ1 (green) in iPSC derived neurons and microglia. Nuclear/cytoplasmic masks for analysis of protein location are represented in rainbow colours (n≥2 cell lines per genotype, n=2 differentiations per cell line, mean ± S.E.M.). Scale bar = 50 µm.

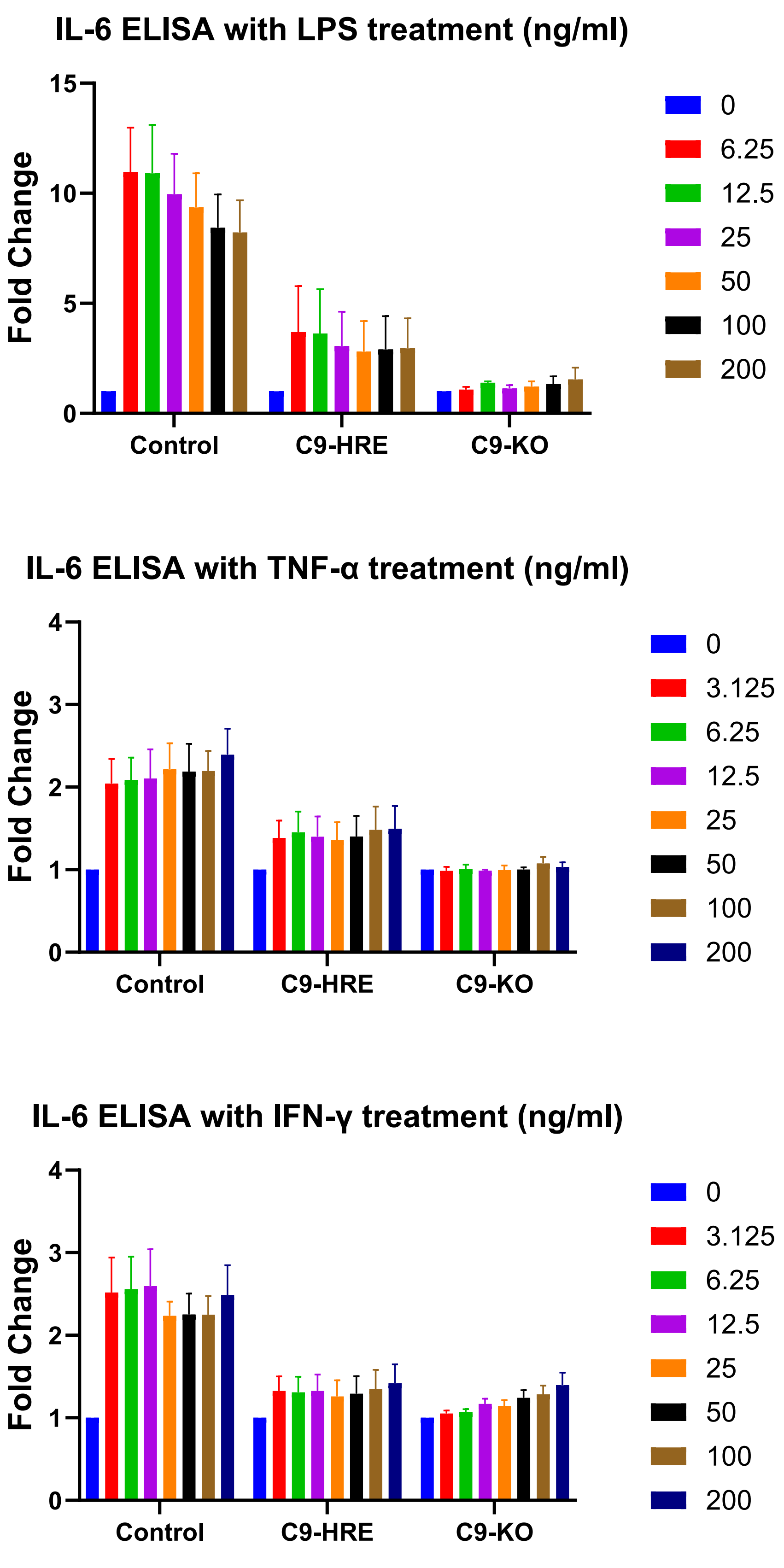

Figure S3a Assessment of IL-6 secretion from LPS, TNF-α or IFN-γ stimulated iPSC derived microglia. Assessment of IL-6 secretion from LPS, TNF-α or IFN-γ (dilution range) stimulated compared to UTC iPSC derived microglia measured by ELISAs (n=2 cell lines per genotype, n=2 differentiations per cell line, mean fold change ± S.E.M.).

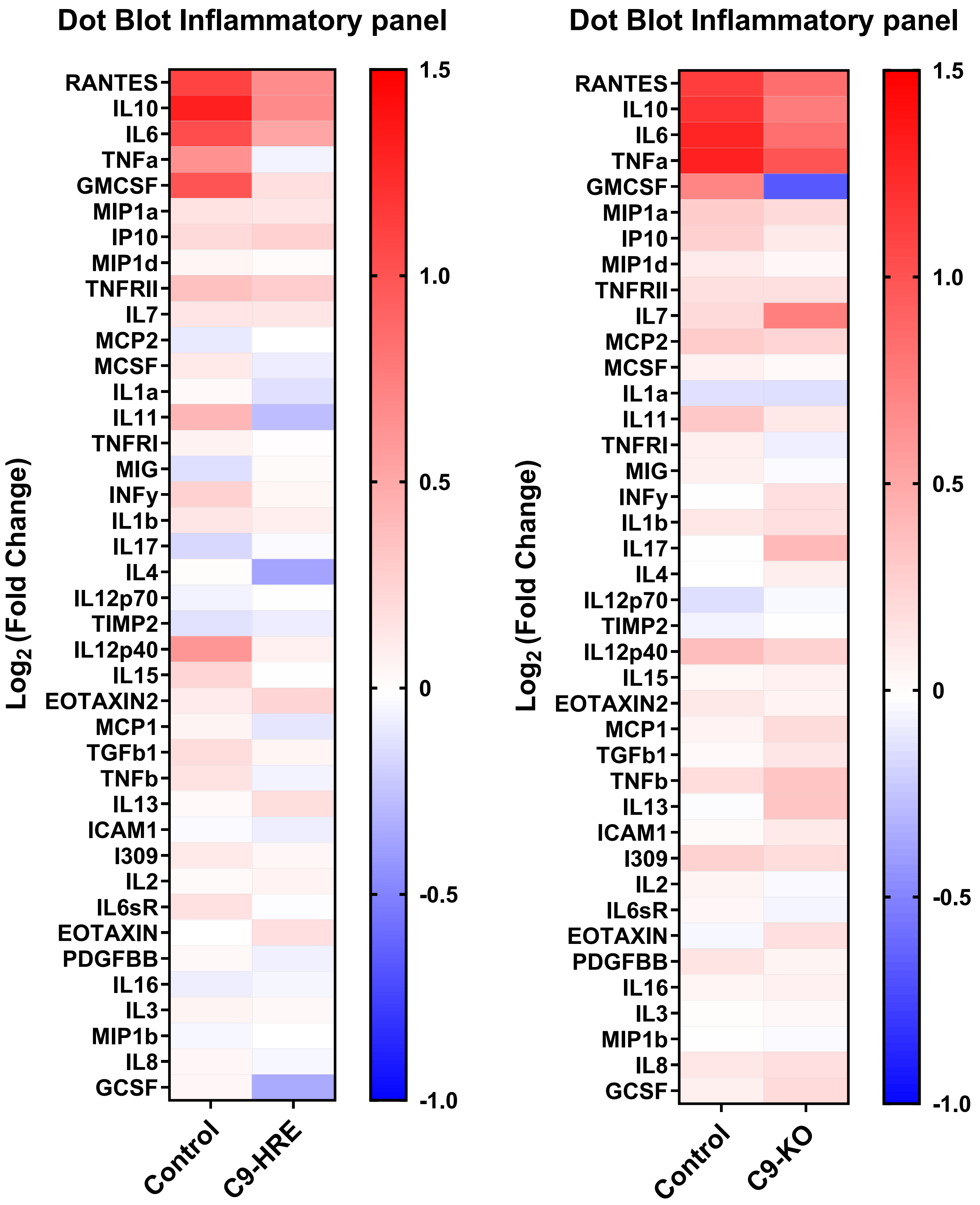

Figure S3b Dot blot inflammatory panel.

Heat maps of log_2 fold change of 40 inflammatory cytokines secreted from LPS stimulated compared to UTC iPSC derived microglia assessed by dot blot assay (n≥2 cell lines per genotype, n≥1 differentiation per cell line, mean log_2 fold change).

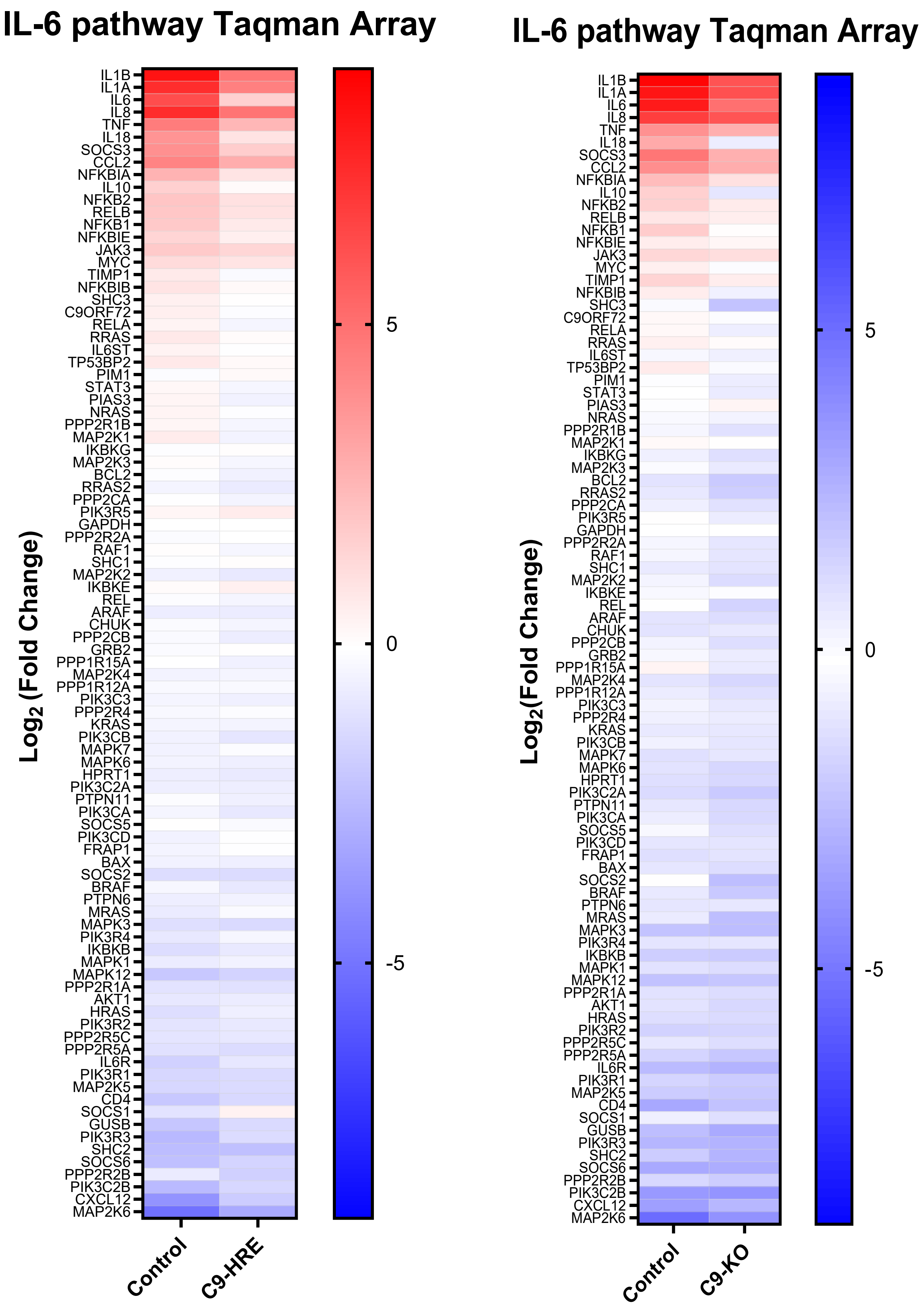

Figure S4 IL-6 pathway Taqman Array.
Heat maps of $\log_2$ fold change of the expression of 92 genes associated with the human IL-6 signalling pathway in LPS stimulated compared to UTC iPSC derived microglia measured by qRT-PCR (n=2 cell lines per genotype, n≥2 differentiations per cell line, mean $\log_2$ fold change).

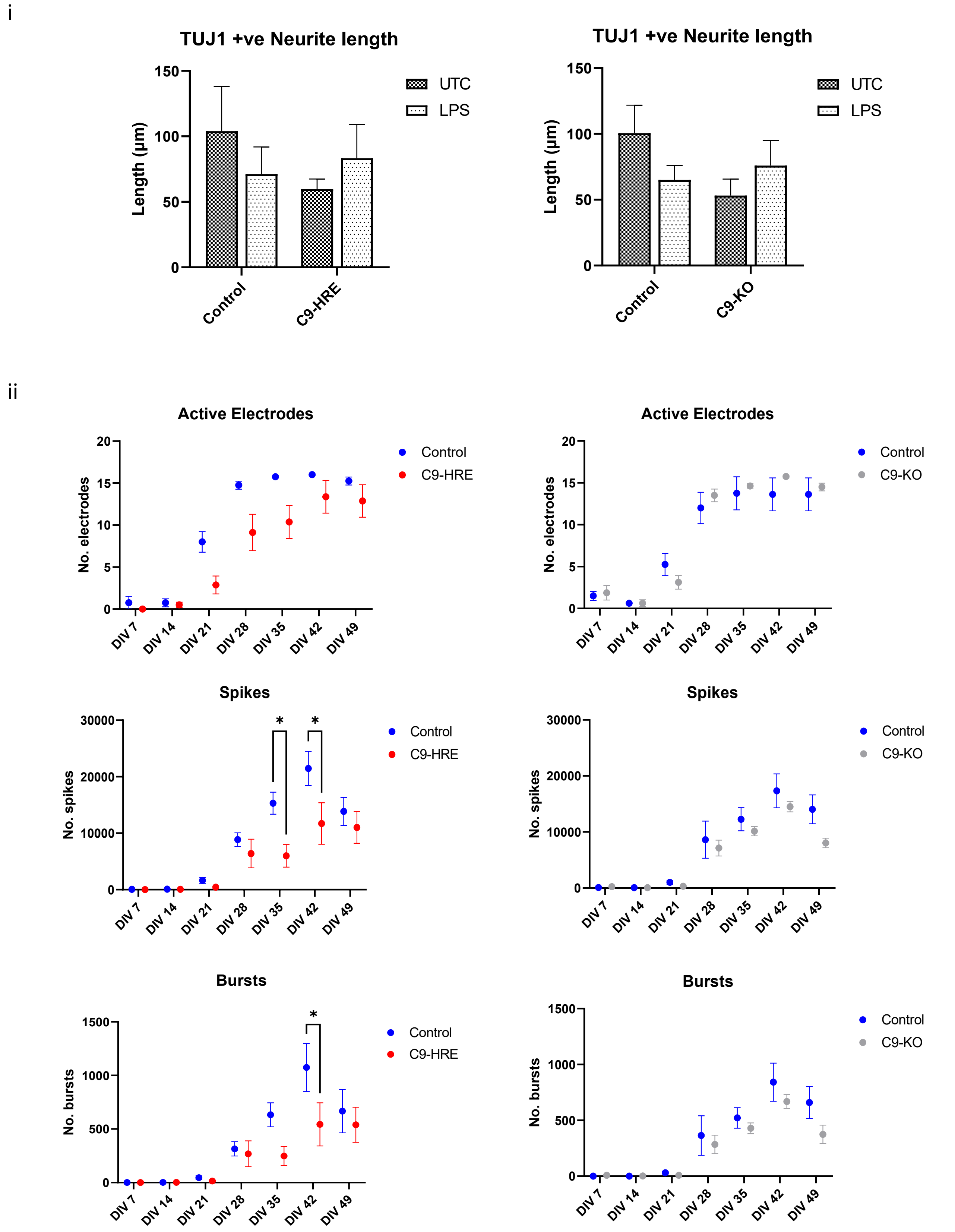

Figure S5 Assessment of neurite outgrowth and electrical activity in iPSC derived neurons co-cultured with microglia. (i) Assessment of neurite outgrowth by TUJ1 immunostaining of neuron/microglia co-cultures (n=2 cell lines per genotype, n=5 differentiations per cell line, mean ± S.E.M.). (ii) The number of active electrode, spikes and bursts detected from neurons cultured with astrocytes by MEA (n=2 cell lines per genotype, n=2 differentiations per cell line, mean ± S.E.M. *p<0.05 in two-way ANOVA test).

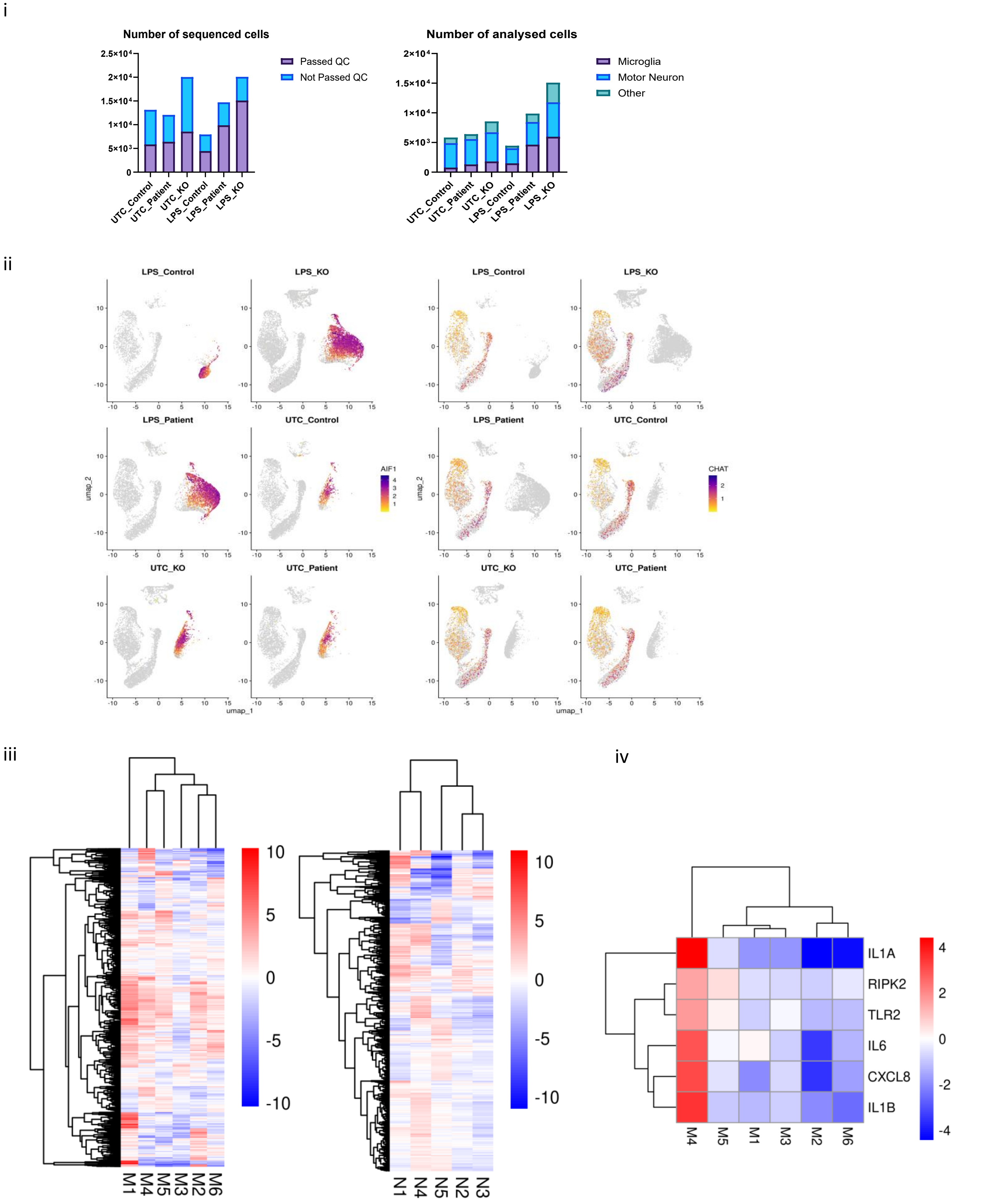

Figure S6a Single cell RNA sequencing.

(i) The number of single cells sequenced and analysed per sample and (ii) UMAPs demonstrating expression of *AIF1* and *CHAT* in distinct cell clusters across samples ($\log_2$ UMI count). (iii) Heatmaps showing average $\log_2$ fold change for 2000 variable features between cell clusters across microglial (M1-6) or neuronal (N1-5) populations described in Figure 6c. (iv) Heatmap showing altered expression on an inflammatory gene set (*IL1A, IL1B, CXCL8, TLR2, RIPK2* and *IL6*) in each microglia cell cluster (M1-6) compared to all other microglia cell clusters.
